# The preparation of carbon nanofillers and their role on the performance of variable polymer nanocomposites

**DOI:** 10.1080/15685551.2019.1565664

**Published:** 2019-02-22

**Authors:** Soad Z. Al Sheheri, Zahra M. Al-Amshany, Qana A. Al Sulami, Nada Y. Tashkandi, Mahmoud A. Hussein, Reda M. El-Shishtawy

**Affiliations:** a Chemistry Department, Faculty of Science, King Abdulaziz University, Jeddah, Kingdom of Saudi Arabia; b Polymer Chemistry Lab. 122, Chemistry Department, Faculty of Science, Assiut University, Assiut, Egypt; c Dyeing, Printing and Textile Auxiliaries Department, Textile Research Division, National Research Centre, Cairo, Egypt

**Keywords:** Carbon-based nano-fillers, polymer nanocomposites, carbon nanotube, graphene, fullerene, graphite

## Abstract

New synergic behavior is always inspiring scientists toward the formation of nanocomposites aiming at getting advanced materials with superior performance and/or novel properties. Carbon nanotubes (CNT), graphene, fullerene, and graphite as carbon-based are great fillers for polymeric materials. The presence of these materials in the polymeric matrix would render it several characteristics, such as electrical and thermal conductivity, magnetic, mechanical, and as sensor materials for pressure and other environmental changes. This review presents the most recent works in the use of CNT, graphene, fullerene, and graphite as filler in different polymeric matrixes. The primary emphasis of this review is on CNT preparation and its composites formation, while others carbon-based nano-fillers are also introduced. The methods of making polymer nanocomposites using these fillers and their impact on the properties obtained are also presented and discussed.

## 1. General introduction

**Figure 1.**
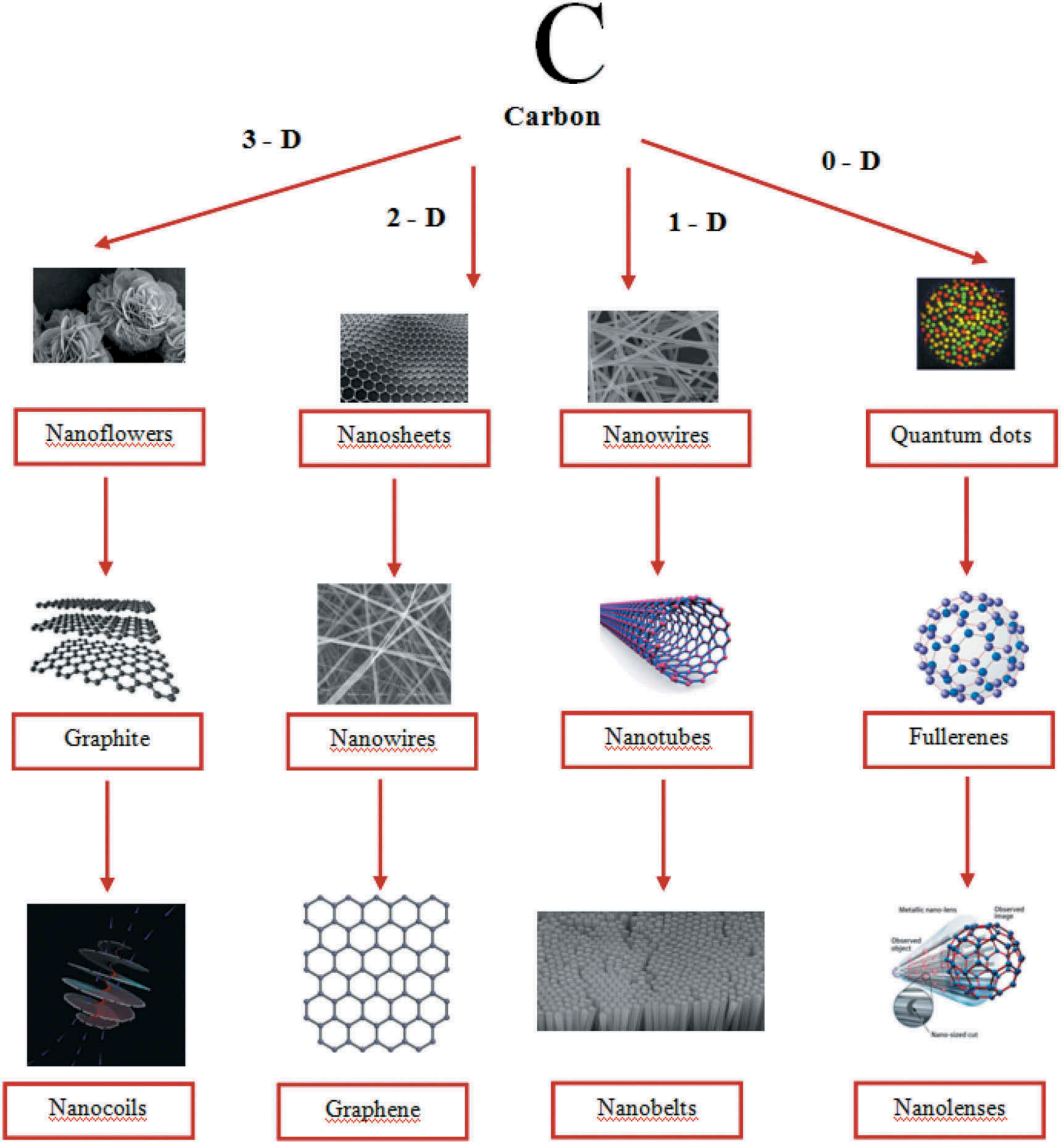
Various forms of carbon and derived structures.

A Carbon is one of the versatile elements among different elements in the periodic table and offers matchless traits. Carbon bureaucracy for the insulator diamond, layered semiconductor graphite, nano-sized fullerenes, the excessive floor place amorphous carbon, and nanotubes are relying upon atomic arrangement and hybridization case. Figure 1 shows the diverse allotropic types of carbon and derived nanostructures.

Polymers are class of compounds typically made up of several repeating units known as monomers. Polymers can be natural or synthetic. Natural polymers are proteins, enzymes, DNA, etc., and some examples of synthetic polymers are polyethylene, polyester, polyvinyl chloride, etc., which we know in the form of fabrics, plastic bags, plastic materials to name a few. Synthetic polymers had revolutionized our world in an unimaginable way in the past century. The material and technological advancements made by synthetic polymers have not stalled yet. Novel materials are being produced every day to meet the needs of humans and address various pressing problems of our society, in which, polymers play a crucial role. Polymers possess excellent bulk mechanical properties, toughness, and viscoelasticity to name a few. Additionally, because of its lightweight, it substituted metals in several routinely used equipment, automobiles, houseware, etc., and it is very resistant to several chemicals. With all these advantages, it is very cost-effective too. Composites are materials usually made up of two or more components. The composite may have superior physical and chemical properties than the individual components that made it. In composites, usually the major components are known as matrix, and one or more minor components play the role of fiber which is known as a reinforcement material. The material used for reinforcement typically imparts the special characteristics or enhancements to the matrix. In certain instances, synergism is seen, where the matrix and reinforcement material complement each other for their exceptional properties. If one or more components in the composite have its dimensions less than 100 nm, such material is known as a nanocomposite. The enhancement of properties of interest in nanocomposites engenders from high surface to volume ratio or aspect ratio or combination of both matrix and reinforcement material. Owing to nanodimensions of components in nanocomposites, even the small amount of reinforcement material significantly influences the macroscopic properties with unforeseen flexibility and sometimes possess multifunctional behavior. If reinforcement materials are in micro or macro dimensions, then the enhancements achieved using them as filler are little to none. Additionally, due to micro or size of filler material traditionally has a higher density which leads to heavier composites which limit its use in several applications. It is interesting to note that originally the employment of filler material for the formation of the composite was used in an aim to reduce the cost of polymeric materials. Later, it was realized such filler materials had reinforcing mechanical properties. Nanocomposites are of three types: (1) ceramic matrix, (2) metal matrix, and (3) polymeric matrix. Our interest will focus on the third type in which the polymeric materials represent the matrix. We will discuss some important examples of a variable polymer matrix reinforced by CNT for variable applications. Accordingly, it was envisioned that conducting such kind of review article to report the impact of carbon nanotubes together with other different carbon-based materials as nanofillers on the properties of different types of polymers would be of great interest for the society of polymers and material science. From our point of view and based on what the literature says regarding this interest, we believe that the present work is more specific and comprehensive in the subject of the study. Hence, the present work deals with the role of carbon nanotubes as well as other different types of carbon materials on the performance of variable polymer nanocomposites.

## 2. Carbon nanotube

### 2.1. Background

The element carbon known since ancient times is of immense usefulness to the mankind through a wide range of applications. Beyond applications, carbon is the building block of every life that we know in this earth as genetic materials, proteins, fats, etc. The allotropic forms of carbon are graphite, diamond, fullerenes﻿ and carbon nanotubes (CNT). Each allotrope of carbon has a distinct and diverse range of physical properties. The summary of the physical properties of allotropes is presented in Table 1. Interestingly, all allotropes of carbon are fairly stable under environmental conditions.

**Table 1.** Physical properties of allotropes of carbon.

Carbon Allotropes Properties	Graphite	Diamond	Fullerene	CNT
Specific gravity (g/ cm^3^)	1.9–2.3	3.5	1.7	0.8–1.8
Electrical conductivity (S/ cm)	4000^p^, 3.3^c^	10^−2^–10^−15^	10^−5^	10^2^–10^6^
Electron mobility (cm^2^/ (V.s))	~ 10^4^	1800	0.5–6	10^4^–10^6^
Thermal conductivity (W/ (m.K))	298^p^, 2.2^c^	900–2320	0.4	2000–6000
Coefficient of thermal expansion (K^−1^)	−1 × 10^−6p^, 2.9 × 10^−5c^	(1 ~ 3) × 10^−6^	6.2 × 10^−5^	Negligible
Thermal stability in air (°C)	450 ~ 650	<600	<600	>700

p: in-plane; c: c-axis

While all allotropes of carbon have unique applications, CNT has gained significant attention in recent decades. The utility of this material was felt in every division of science and found a variety of applications. CNT influence in science and technology was a combination of two factors: (1) properties of the material unique to itself and (2) nanodimensions. The vital reason and all the attraction nanotechnology has received is essentially due to the vast difference in property of material when compared to its atomic and bulky state. Nanoscience is the study of the property of materials at dimensions in the range of 1–100 nanometer (nm). Size effects have a profound influence in the mechanical properties of materials which is in unswerving relationship to electrical and other chemical properties of the materials. Strength, elasticity, plasticity, toughness, and hardness of the materials are considered as mechanical properties of the nanomaterials. The significant difference of mechanical properties of nanomaterials from its bulky or atomic states is due to the surface or interface energies and stresses. The unique properties of nanomaterials arise from the greater surface area to volume ratio, high surface energy, spatial confinement, and new quantum effects. At the macroscopic scale, i.e., in millimeter scale, the number of atoms exposed to the surface is typically in negligible proportions compared to the total number of atoms in the bulk material. Hence, the surface atoms do not affect the bulk properties. However, in nanodimensions, the proportion of atoms exposed to the surface cannot be ignored. Thus, the nanodimension of carbon enhances its physical properties and makes it unique and interesting.

Of the various forms of carbon in the nanodimension, we mainly devote our attention to CNT. The interest in the CNT was triggered after the publication from Iijima

[1] in 1991, even though it is not the first report on this material. The interest in that publication was largely due to a detailed characterization that led to identifying the exceptional properties of the CNT and identification of potential applications. CNT exists in two forms: (1) Single-Walled Carbon Nanotube (SWCNT) and (2) Multiwalled Carbon Nanotube (MWCNT). The CNT reported in 1991 publication was MWCNT. SWCNT was discovered independently by research groups of Iijima [2] and Bethune [3]. SWCNT is a single layer of graphene rolled into a cylinder whereas, MWCNT is concentric cylinders of graphene. The prime difference between the SWCNT and MWCNT is summarized in Table 2[4].

**Table 2.** comparison of SWCNT vs MWCNT.

SWCNT	MWCNT
Single layer of graphene. Catalyst is required for synthesis. Bulk synthesis is difficult as it requires proper control over growth and atmospheric condition. Not fully dispersed and formed bundled structure. Resistivity usually in the range of 10^−4^–10^−3^Ωm Purity is poor. Typical SWCNT yield of as-prepared samples by chemical vapor deposition (CVD) method is about 30–50 wt %. However, high purity up to 80% has been reported by using arc discharge synthesis method. A chance of defect is more during functionalization. Characterization and evaluation are easy. It can be easily twisted and are more pliable.	Multilayer of graphene. Can be produced without a catalyst. Bulk synthesis is easy. Homogeneously dispersed with no apparent bundled formation. Resistivity usually in the range of 1.8 × 10^−5^–6.1 × 10^−5^ Ω. M Purity is high. Typical MWCNT yield of the as-prepared sample by the CVD method is about 30–90 wt%. A chance of defect is less especially when synthesized by arc discharged method. It has a very complex structure. It cannot be easily twisted.
	

**Figure 2.**
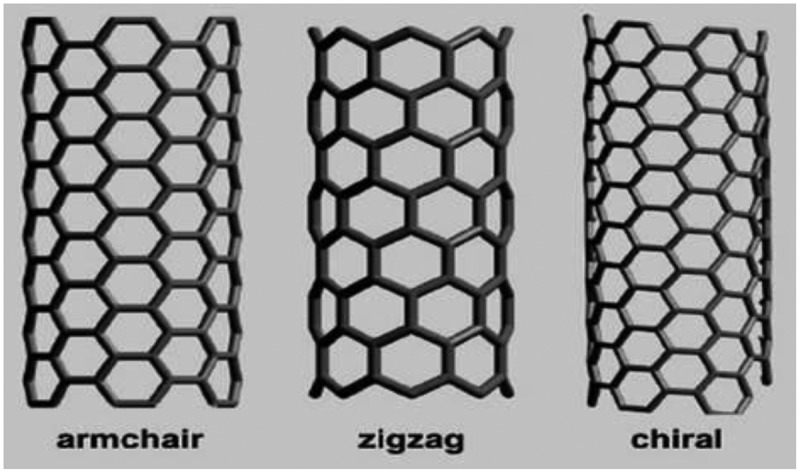
Scheme depicting the different conformations of SWCNT.

SWCNT is classified into three types based on its crystallographic configurations as zig-zag, armchair and chiral as shown in Figure 2 [5]. This classification is interpreted upon how the graphene sheet is rolled up to form the SWCNT. When graphene sheet is folded, if the opposite carbons coming together to form cylinder and the c-c bonds were in the axis of the cylinder, it is called zigzag confirmation; however, if the opposite c-c bonds lie perpendicular to the axis of the cylinder, then it is known as arm-chair conformation. If the opposite c-c bonds lie in an angle to the axis of the cylinder, then the conformation is known as a chiral nanotube. It should be noted that SWCNTs of any conformation mentioned above are not synthesized from graphene sheets; for sake of understanding purpose only, it is explained using graphene sheet.

Properties of carbon nanotubes: the properties of CNTs are associated with the type of chemical bonds between the carbon atoms in the nanotube. As CNTs can be visualized as graphene sheets rolled up to form the structure and graphene sheets are simply a layer of graphite, thus, we can conclude that carbon is in sp^2^ hybridization. The exemplary stiffness and strength of the CNTs are associated with stronger covalent sigma between the carbon atoms in graphite that has bond energy of 346 KJ/mol. The CNTs are approximately six times lighter than stainless steel and Young’s moduli measure more than 1 TPa which makes it at least five times stiffer than steel [6]. The strength of the CNTs are highest of any material known so far, the measured tensile strength was up to 63 GPa [7]. To put it perspective, the measured tensile strength was approximately 50 times more than that of steel. In addition to stiffness and strength, CNTs have a negligible coefficient of thermal expansion, very stable under environmental and chemical conditions and high thermal conductivity. Summary of the properties of CNTs mentioned here is presented in Table 3 [8]. Combined superlative properties of CNTs mentioned above make it most sought material for a variety of applications and innovations.

**Table 3.** Summary of properties of CNTs.

Mechanical properties	(1) Young’s modulus of SWCNTs ~ 1 TPa (2) Young’s modulus of MWCNTs ~ 1–12 TPa (3) Tensile strength of SWCNT ropes ~60 GPa Tensile strength of MWCNT ~0.15 TPa
Thermal properties at room temperature	Thermal conductivity of SWCNT ~ 1750–5800 W/mk Thermal conductivity of MWCNT > 3000 W/mk
Electronic properties	SWCNT bandgap When n-m is divisible by 3(0 eV, metallic) When n-m is not divisible by 3(0.4–2 eV, semiconducting) MWCNTs bandgap ~0 eV (non-semiconducting)
Electrical properties	Typical resistivity of SWCNT and MWCNTs = 10^−6^ Ωm Typical maximum current density = 10^7 −1^0^9^ Acm^−2^ Typical quantized conductance (measured) = 12.9 kΩ^−1^

### 2.2. Synthesis of CNTs

**Figure 3.**
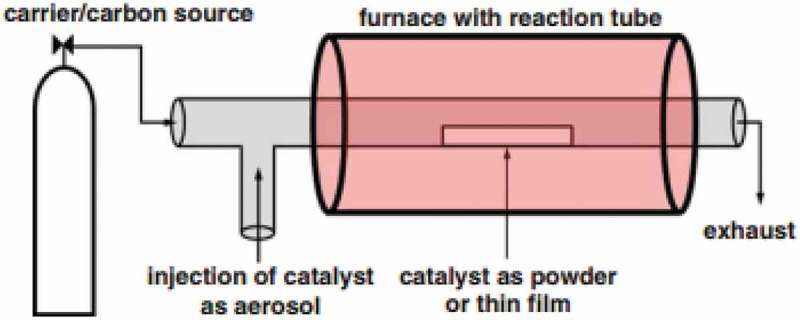
Generic experimental setup scheme for the synthesis of CNT by CVD (adapted from reference [15]).

As CNTs were found to be a suitable material for a variety of applications, each application requires certain characteristic or feature in CNTs, such as high aspect ratio, chirality, pore diameter, alignment, shape, and dispersion to name a few. Apart from such properties, it is also desired to be able to produce it in large quantities in a reproducible method. CNTs with desired properties can be achieved by synthesis methods, tuning reaction parameters from the source of carbon, catalyst, substrates, etc. The strategies for the synthesis of CNTs are: (1) chemical vapor deposition, (2) laser ablation (3), arc discharge, (4), electrolysis, and (5) flame synthesis. However, the first three methods are most explored or adopted as it was advantageous over other methods. Chemical vapor deposition (CVD): CVD is the most investigated method for the synthesis of CNTs as it offers better control, straightforwardness, bulk production, simplicity, and cost-effective [9,10]. Owing to its popularity, several reviews for the synthesis of CNTs were published in literature regularly highlighting the recent developments achieved in the synthesis [11–14]. In a typical synthesis, the precursor of carbon will be gasified and flown using a carrier gas into the reaction chamber containing a substrate to be coated. The generic scheme of the CVD reactor is shown in Figure 3. The reaction occurs near the hot surface followed by condensation into a thin film on the catalyst that is previously deposited on the surface of the substrate. Due to the high temperatures employed in the synthesis, the gasified carbon precursor in the atomic state approaches the substrate surface and fills the cavities of the substrate to form a solution of metal-carbon. Upon supersaturation of the substrate, the precipitation of carbon begins triggering nucleation for the development of nanotubes. The catalyst on the surface of the substrate encapsulates the nanotube either on top or bottom of the substrate depending on the growth mechanism. There are several variables associated with the synthesis of CNTs that can be manipulated to achieve the desired features depending on the applications of CNTs. The tunable parameters are flow rates of carrier gas, nature of the substrate, a carbon precursor, temperature, reaction time, nature and quantity of catalyst, the concentration of precursor, etc.

Next, selective parameters mentioned above will be discussed briefly from the recent literature (within past 10 years) to understand its influence on final CNT product.

#### 2.2.1. Role of the precursor

A variety of precursors were explored for the synthesis of CNTs. Typically, they are hydrocarbon-based chemicals such as ethylene, acetylene, methane, benzene, xylene, toluene, cyclohexane, and petroleum-based products. In addition to that, polymers such as polyacrylonitrile and polyfurfuryl alcohol carbon monoxide, and alcohols were explored too [8]. With increasing environmental concern, recently, synthesis of CNTs utilizes environmental friendly precursors such as camphor [16], wastes generated oil and petroleum fields, turpentine oil [17], asphalt (de-oiled) [18], coconut oil [19], palm oil [20], neem oil [21], eucalyptus oil [22], and cooking waste oil [23]. This is a comprehensive list of precursors explored for the synthesis of CNTs via CVD and any means it is not a complete list of precursors investigated until now. Carbon precursor influences growth, characteristics, and properties of CNTs owing to its thermodynamic properties, binding energy, etc. The growth of CNTs from gaseous carbon precursors is influenced by the concentration of precursor and intermediates produced in the gas phase along reactive radical species generated during the pyrolysis of hydrocarbons. During the pyrolysis, the intermediates produced should be able to stick to the catalyst surface via physisorption or chemisorption to initiate the growth process [24]. Saturated hydrocarbons have the preference to produce SWCNT, and unsaturated hydrocarbons have the ability to form MWCNT [25,26]. Alcohols were investigated as precursors for the production of CNTs with a wide variety of catalyst. It is widely observed that alcohols are good for production of SWCNT that is amorphous carbon free at relatively low temperature than hydrocarbons. This is due to the etching effect of hydroxyl radical generated from ethanol. In other words, steam acts as a mild oxidizer for efficient removal of CNT. In addition to amorphous-free CNT production from ethanol, it can produce vertically aligned SWCNT when acetylene was supplied intermittently [27,28]. Ultralong CNTs were produced with ethylene precursor using water and hydrogen [29]. By employing an appropriate choice of the carbon precursor and optimal feed rate of water high-quality CNTs can be produced in good yields [30,31]. Hydrocarbon precursors such as benzene, acetylene, and xylene to produce MWCNTs in high purity are not ideal for high-temperature synthesis. Under temperatures between 1073 and 1473 K, the abovementioned precursors become highly unstable leading to the formation of amorphous carbon. The ideal precursors for high-purity production of CNTs for high temperatures, CO, and methane are preferred. It should be noted that under high temperatures, SWCNTs form preferentially than MWCNTs and vice versa [32,33]. LPG has been explored as precursors as an alternative to conventional hydrocarbon precursors for the production of CNTs in bulk. Several research groups published with this precursor recently [34–36]. For this precursor with NiO catalyst, an average diameter of 25 nm was synthesized [37]. Pasha et al. studied various catalysts for LPG precursor for the production of CNTs. Fe-Cr catalyst produced CNTs with a wide pore size distribution between 51 and 169 nm at variable temperatures between 850°C and 1050°C [38]. Palladium catalyst at temperatures between 630°C and 800°C yielded CNTs with diameters ranging from 39 to 109 nm [36] and Ni-Si catalyst produced CNTs with diameters of 37 nm [39]. It is evident from this observation that by careful choice of reaction conditions and catalyst, the CNTs pore size can be manipulated.

Compressed natural gas was also explored as a cheap alternative precursor for the production of CNTs. Koskatova et al. produced CNTs with a wide variety of unconventional substrates ranging from rocks, metal plates, and wires. CNTs were produced along with other nanostructures. Cuprothal and Nikrothal wires produced relatively long MWCNTs at temperature 800°C at a flow rate of 40 mL/min [40]. Lee et al. reported the use of CNG as a precursor; CNTs were produced and found that yields were relatively larger than purified methane gas. Side products with CNTs were reported by them, and the growth mechanism of side products were not explained [41]. Heavy petroleum fractions such as light diesel oil and furnace oil are petroleum byproducts [42]. The soot collected from this precursor using a specially designed reactor was investigated for dispersion in various solvents. Acetone and water demonstrated better dispersion. The diameters of the CNTs were reported between 70 and 90 nm. Heavy oil residues were investigated by Li et al., and SWCNTs were synthesized. Magnetic metals such as Fe, Co, and Ni were explored as a catalyst along with non-magnetic noble metals Au and Pt. Magnetic metal catalysts yielded narrow pore size distribution than noble metals. Choice of catalyst and temperature was a key for the production of SWCNTs [43]. Waste engine oil is explored as a carbon precursor that offers environmental and economic benefits [44]. Quasi-aligned CNTs were formed owing to its large carbon content. Ferrocene was used as a catalyst, and temperature between 500°C and 750°C was explored. CNTs formed found to be a mixture of MWCNT and SWCNT. The complex chemical composition of engine oil and contaminants in it had negligible influence on the outcome of CNTs. Growing environmental concerns triggered several strategies to be evolved from various renewable resources as CNT precursors. Paul et al. explored coconut oil as CNT precursor using Fe catalyst. MWCNTs were produced under the reaction conditions, and the diameters were in the range of 80–90 nm under the optimal synthesis conditions. The lauric acid and oleic acid is unique to coconut oil which cannot be found in other oil precursors [19]. Fathy investigated camphor as the precursor of CNT through carbonization of rice husk. Mono-catalyst of Fe and catalyst mixture of ferrocene and Ni were utilized. Coiled CNT-Fe bundles were produced on carbonized straw for ferrocene catalyst with smaller outer diameters. For mixed catalyst, reasonable amounts of CNTs with straight and larger outer diameters were produced [16]. Palm oil was used as a precursor for the synthesis of CNTs by Suriani et al. [45]. Vertically aligned CNTs were synthesized that is a dense mixture of MWCNT and SWCNT. The SWCNT had a diameter of 0.6 nm–1.2 nm with 90% purity. Recently, several review articles were published for the synthesis of CNTs and related materials from sustainable natural resources to waste from industry poultry, etc. It should be noted that some of the synthesis strategies listed in the reviews may be beyond CVD [46–48].

#### 2.2.2. Catalysts in CNTs CVD synthesis

The final product quality and yield of CNT is a function of every reaction component used in the synthesis and reaction parameters. It ranges from the method of synthesis, precursor, substrate, catalyst, temperature, flow-rate, reaction time, carrier gas, to name of a few. Of all the various parameters, we can imagine influencing the CNT formation, purity, quality, and yield catalyst plays the crucial rule. In fact, the catalyst is assumed as the father of the resulting nanotube. The catalyst employed in the synthesis plays the role of nucleation sites. The need and role of catalyst in synthesis of CNT summarized by Jourdain et al. [11] are as follows: (a) decomposition of CNT precursor at a temperature lower than its spontaneous combustion to form elemental carbon; (b) permissible diffusion of carbon intermediates and its interactions; (c) serve as template for nucleation and growth of CNT; and (d) availability of reactive nanotube rim. SWCNT synthesis is highly dependent on catalyst; however, MWCNT can be grown without the catalyst. It is not just the catalyst influences the CNT formation; in fact, the preparation method of the catalyst also found to influence the CNT growth and formation. For example, the number of walls in the final product is highly dependent on the right selection of catalyst. The right catalyst selection for the synthesis of CNT with desired properties should be based on one or a combination of several parameters [49]. The parameters are: (1) nature of the catalyst, (2) method of preparation, (3) concentration to be employed, (4) textural properties of catalyst such as pore size, pore diameter, (5) pore size of the support, (6) catalyst nanoparticle dimension, (7) melting point, (8) promoter, and (9) metal support interaction, etc.. Conventional catalysts for the synthesis of CNT by CVD are the transition metals iron, cobalt, and nickel. The reason behind such common utilization of these metals as catalysts is due to finite solubility of carbon in those metals and superior diffusion rates. Apart from that, these metals have low equilibrium vapor pressure over a broader temperature range of CVD which gives the flexibility to use various carbon precursors. The adhesive forces between the growing CNT and the conventional catalysts are relatively stronger which aides in the efficient formation of high-curvature SWCNT [50,51]. The general trend is higher the d-orbital vacancies, better the solubility of carbon. Thus, Fe, Ni, and Co have finite solubility, early transition metals such as Ti, Mo have very high solubility of carbon, and elements such as Zn and Cu which has completely filled d-orbitals have a negligible affinity to carbon. The CNT formation will be achieved regardless of the catalyst only when there is enough carbon beyond the solubility of carbon in metal catalyst. Thus, graphitic carbon will be formed more readily for catalyst metal with low solubility of carbon than the metals that have high solubility with carbon.

The exploration of catalysts for CNT synthesis extends beyond conventional Fe, Co, Ni catalysts. Other metallic catalysts investigated are Cu, Ag, Al, Cr, Mo, Mn, Pd, and Au; metalloids such as Si and Ge, and nonmetallic diamond were also used as catalysts for CNT synthesis [52–55]. In a report by Hu et al., they have used Fe and Al catalysts; however, they were released gradually to produce high-density SWCNT [56]. They named this catalyst as Trojan Catalyst; in analogy with Greek story, the catalyst precursors were hidden in the substrate, and under the reaction conditions, the catalysts were released from the substrate and reduced to active catalyst form. Conventional Fe, Co, Ni catalysts were investigated as binary mixtures of active catalysts and observed to be more active than individual catalyst nanoparticles[57]. In another strategy, promoters such as Mg and Mo were mixed with traditional catalysts. The promoters tend to prevent the coarsening of active catalyst nanoparticles [11,58].

### 2.3. Polymer nanocomposites reinforced CNT

#### 2.3.1. Polyethylene CNT nanocomposite

CNTs in biomedical and pharmaceuticals application have been of concern owing to the possibility of being incompatible and/or cytotoxic for the human cells. The common understanding is that CNTs regardless of size in functionalized and unfunctionalized form are not ideal [59–61]. The non-suitability of the CNTs for such applications is due to the size of the CNT, agglomeration, impurities in it as the major reasons. Ultra-high molecular weight polyethylene (UHPE) is commonly used in biomedical applications owing to its ideal physicochemical properties [62,63]. UHPE that in linear chain form is desired for biomedical applications; however, its low strength limits certain applications. To overcome this challenge, Mamidi et al. had UHPE reinforced with MWCNT and cytotoxicity was assessed with human fibroblast cells [64]. A series of UHPE/MWCNT nanocomposite with MWCNT ranging from 0.05 to 5 wt % was prepared by ultrasonication followed by hydraulic pressing. Such prepared nanocomposites were investigated by DSC analysis; it indicated there is no interaction between MWCNT and UHPE. The crystallinity was most improved for 5 wt %. The presence of MWCNT increased the roughness of the fracture surface as identified by SEM analysis. Non-homogeneous distribution of MWCNT was found at low wt %, and it improved to homogeneous dispersion at higher wt % with negligible accumulation. The mechanical properties tensile strength, Young’s modulus, and yield strength of the nanocomposites were significantly improved than the pure UHPE. The roughness profile of the nanocomposite increased with increase in MWCNT content. The cytotoxicity of MWCNT was measured with human fibroblasts with varying concentration of nanocomposites. The confocal images obtained after 72 h of incubation revealed the cell growth was increased with increasing the weight percent of the MWCNT suggesting a positive influence of MWCNT in fibroblasts. The contributing factors for the growth of fibroblast were surface morphology, the contact angle with cell media, and surface chemistry of the composite. Quantitative cell viability studies of nanocomposites were higher than pure UHPE at 24 h, and good proliferation was observed up to 48 h.

For polymer nanocomposite to exhibit superior properties than pristine polymer, it is a strong adhesion or coupling between filler and polymer is desired [65–68]. Functionalization of CNTs is the key to instill such strong adhesion. The strategies to have them in the nanocomposites are conventionally by chemical, plasma, and electrochemical methods. Chouit et al. have synthesized CNT functionalized with nitrogen via CVD using alumina as substrate [69]. Such synthesized CNTs having 40 nm diameter were used in various wt % to reinforce high-density polyethylene (HDPE) via melt mixing method. The distribution of filler was through shearing action. The nanocomposite was found to be 97% pure with bamboo-like structure. The presence of filler did not affect the crystalline structure of HDPE. If the CNTs with high aspect ratio were used as filler, it is considered to have enhanced magnetic resonance imaging (EMI) shielding and smaller electrical percolation threshold. But the downside is the cost of CNTs with high aspect ratio which are expensive; hence, it is necessary to find strategies to decrease the filler amount to make it cost-effective. UHPE/CNT nanocomposite was synthesized by wet mixing under ambient conditions by Al Saleh [70]. This synthesis method yields nanocomposite with filler selectively occupying the external surface that has CNT free zone around 20–50 times larger than the nanocomposite prepared by conventional techniques such as melt mixing or solution processing. Optical microscopy characterization of the composite revealed CNTs are externally coated with continuous networks. A linear relationship was found for the thickness of the CNT coating with a varying weight percent of CNT. The electrical percolation threshold was found at 0.054 vol % at 0.096 wt % of CNT. The control synthesized using dry-mixed nanocomposite electrical resistivity above percolation was lower by 2–3 order magnitude. The EMI shielding effectiveness increased with the increase in CNT wt %, and highest was observed for 10 wt % at 50 dB which is very much higher than reports published in literature prior to this.

Ahmad et al. studied the effect of functionalization of CNT on rheological properties of polyethylene nanocomposites [71]. Two series of HDPE CNT composite were prepared with filler wt % starting from 1 to 7. In one series, pristine CNT was used as filler; in another series, functionalized CNTs (C18 modified and phenol modified) were employed. Both of them prepared using melt blending process. At higher wt % of nanofiller, the hydrodynamic effect was observed explicitly and nanocomposite viscosity increased in a sharp manner regardless of the fact that CNT was functionalized or not. Viscosity was decreased in lower wt % nanocomposite in both forms of nanofiller (functionalized and pristine) due to the increase in disentanglement of polymer chains. The magnitude of the drop in viscosity of functionalized CNTs is more than pristine. The order of viscosity drop for 3 wt % loading is pure polymer <C18 modified < phenol modified < CNT, and this magnitude of difference is enhanced at higher loadings. The steady shear viscosity and storage modulus of HDPE also showed the similar trend at a loading of nanofiller at low to medium wt % of CNT loading. High loading wt % nanofiller enhanced the above properties in a significant fashion due to filler network formation in the matrix. The percolation threshold is 4 wt % for unfunctionalized CNTs and 7% for functionalized CNTs. At lower wt % of nanofiller, the dispersion was achieved well, but at higher wt % some agglomeration was observed.

Polymer nanocomposites containing metal nanoparticles have superior mechanical, electrical, and thermal properties along with flame retardancy to name a few [72–77]. To such nanocomposites, adding CNT will enhance the properties much higher along with multifunctionality. Nisar et al. have synthesized polyethylene nanocomposite reinforced with Fe-encapsulated CNT via *in situ* polymerization to facilitate uniform dispersion. The polymeric nanocomposite had a decrease in degradation temperature than its control without Fe. Such a decrease in degradation temperature is evidential of Fe in nanocomposite which catalyzes this process. SEM analysis suggests that nanocomposite had fibrous morphology. When Fe content was increased in the CNT, the fibrous morphology was more prominent. Additional attribution factor for fibrous morphology was its low polymerization synthesis temperature. Nanocomposite without Fe did not exhibit hysteresis loop, suggesting it is diamagnetic. However, for nanocomposite with Fe, it exhibited hysteresis loops and no significant difference in remnant magnetization values was observed with increase in loading of Fe containing CNT. The coercivity value was dropped due to the increase in Fe-containing CNT content which was attributed to similar mean interparticle distance. The hysteresis loop of nanocomposite recorded for the same sample with a time interval of 6 months was the same, suggesting the CNT prevents the oxidation of Fe and aggregation. Additionally, the polymeric properties of polyethylene have not changed at all and it simply adds up the magnetic property due to the addition of Fe containing CNT which enhances the potential applications of the material where flexible magnetic behavior is desired.

The employability of polyethylene is very limited or unsuitable for applications that have a low operating temperature. Crosslinking of polyethylene enhances the features of polyethylene with respect to abrasion, mechanical performance, and operating temperature to name a few. Thus, crosslinked polyethylene reinforced with CNTs to attain higher polymer thermal conductivity for geothermal application were studied by Roumeli et al. [78]; polyethylene grafted with vinyltrimethoxysilane was mixed with MWCNTs in a solid state centrifugal ball mill followed by melt mixed and hot pressed and immediately cooled. Four nanocomposite samples with wt % of 0.5, 1, 3, and 5 were synthesized. All synthesized samples had specific heat capacity of more than 100% to that of control. Nanocomposite with 0.5 wt % of MWCNT showed the highest specific heat capacity of 150% when compared to neat polymer. This trend was observed in thermal diffusivity and thermal conductivity measurements. TGA analysis of nanocomposites suggested a mild enhancement of thermal stability in the range 6–7°C. However, significant changes in the kinetics of decomposition were observed. The pyrolysis of nanocomposite produced high-molecular weight ions initially and then conventional small hydrocarbons characteristic of polyethylene. The heavier fragments are octamethycyclotetrasiloxane, decamethylcyclopentasiloxane, and dodecamethylcyclo-hexasiloxane which was derived from crosslinking agent poly(dimethysiloxane) that was produced first. Formation of cyclic products was due to activated depolymerization reactions. The molecular ions derived from polyethylene are randomly having 4–10 carbon fragments which are formed later at a later stage of pyrolysis. The delayed release of fragments was due to hindrance from CNTs that suppress the passage of primary scission products of the nanocomposite.

UHPE polymer is most widely used in joint replacement applications (implants) that counteract with surfaces made of metals and ceramics [79,80]. The prevalence of such applications is due to its superior toughness, low friction, impact strength, and abrasive sliding wear resistance along with its excellent biocompatibility [81]. The life span such implants made by these materials is largely determined by its tribological behavior of materials such as low yield strength and severe wear that lead to premature failure [82]. Additionally, when UHPE implants are in relative motion with metal or ceramic material, it generates wear debris which migrates to tissues that cause osteolysis [83]. The shortcomings of UHPE for these types of applications can be improved by reinforcing the UHPE with CNTs. The properties of nanocomposite were superior for desired application when the CNTs are dispersed homogeneously, and interactions between polymer and CNTs are well established [82,84,85]. To achieve that, the aspect ratio of CNT plays a crucial role. Kumar et al. studied the role aspect ratio in the tribological and mechanical behavior of UHPE/CNT nanocomposite [86]. UHPE nanocomposite was prepared with high and low aspect ratios of 900 and 75, respectively, of CNT. For both aspect ratios, 0.05 wt % and 0.1 wt % of reinforcement are used to avoid agglomeration. Hardness and modulus of the UHPE with a high aspect ratio of CNTs are significantly higher as identified by indentation experiments which were attributed to high surface to volume ratio and interfacial strength. Additionally, high aspect ratio composite has more flocks leading to percolation network. Initially, coefficient friction increases with distance and then steady out; however, the friction force for nanocomposite with the high aspect ratio is smaller than PE nanocomposite with low aspect ratio and pure PE, respectively. The width and depth of wear tracks, specific wear rate are significantly lower for CNTs with high aspect ratio. Overall, the PE nanocomposite with a high aspect ratio of CNTs had improved mechanical and tribological properties as toughener and solid lubricant to be employed as joint replacement implants. In another study, Evgin et al. explored the effect of aspect ratio on thermal conductivity of HDPE/MWCNT [87]. The aspect ratios of CNT filler were chosen in the range of 200–400 and 500–3000, to reinforce with HDPE by melt mixing. The nanocomposite with high aspect ratio had high thermal conductivity enhancement than the nanocomposite with low aspect ratio. At low concentrations of filler, *k* enhancement was up to three- to four-fold higher than composite with low aspect ratio. Such direct correlation to thermal enhancement with aspect ratio was attributed to the formation of percolation network that enables the passage of phonons without transitions. The MWCNTs that has smaller diameter was found to be least resistant to mechanical stress.

Gao et al. have studied submerged friction stir processing (SFSP) for the synthesis of HDPE/MWCNT nanocomposite under ambient conditions and studied the morphology, mechanical, and thermal properties of composites with respect to FSP parameters [88]. This method is just a variation of friction stir processing (FSP) where the process takes place at a certain ambient temperature in a stationary water bath or water circulation equipment. In this process, blind holes were put in the HDPE followed by filling of holes with MWCNTs and wrapping the surface with HDPE with no holes of the same length as in the lower layer. The samples prepared by SFSP had roughness lesser than samples prepared with FSP due to lower temperature and high cooling rate under the submerged conditions. At low tool rotational speed, inadequate mixing of HDPE and nanofiller was observed as sufficient heat was not generated. At higher speeds, the macrostructure was homogeneous; however, at very high speed of 2100 r min ^−1^, the nanocomposite turned porous due to decomposition of HDPE in heat. The tensile strength increased by 20% for the sample prepared by SFSP which was higher than the samples prepared by melt mixing and FSP. The high values are due to better dispersion and energy absorbing effect of filler in the matrix. The crystallinity of the nanocomposite was 6% more than the pure polymer. Overall, the SFSP method to prepare the nanocomposite by altering the various reaction conditions, reasonable tuning of properties of the nanocomposite can be achieved. For non-polar polyethylene, breaking down the bundles of CNTs is essential for good dispersion of filler to achieve the enhancement for desired applications. Liu et al. synthesized polyethylene nanocomposites with core-shell structure that prevents agglomeration of filler [89]. They have performed *in situ* Ziegler-Natta polymerization with CNTs as the core and polyethylene as the shell. The MWCNTs were oxidized to introduce the oxygen in the CNT as functional groups such as hydroxyl and carboxylic acids. Upon oxidation, it was treated with methyl magnesium chloride to replace the acidic H’s by magnesium chloride followed by treatment with TICl_4_. Through these steps, the catalyst was supported on CNT and polymerization was performed on the support to get the nanocomposite with core-shell structure. The polyethylene had a similar molecular weight and broader distribution. The CNTs were covered by polyethylene to a thickness of 450–500 nm. The growth mechanism was thought to be polymerization initially at catalytic centers, followed by the formation of polyethylene sleeves due to excessive piling and sliding and the third step was conjoining of sleeves to form a homogeneous coating. Crystallinity decreased due to CNTs than the pure polyethylene; however, no difference in melting temperature was found. The polyethylene in nanocomposite retained all the properties of pure polyethylene. Even though the nanocomposites provided an improvement in thermal stability, the dielectric results did not show significant improvements. The property that limits the application of polyethylene is its ultra-low thermal conductivity. Liao et al. have demonstrated that it can be improved by adding thermally conductive aligned CNTs as fillers through non-equilibrium molecular dynamics simulations [90]. Such improvement was due to reduced phonon scatterings at interfaces of filler and polymer. The nanocomposite with aligned CNTs had thermal conductivities three orders higher than pure polyethylene. The mechanism for enhancement in conductivity was due to the alignment of CNTs avoidance of interface scattering, van der Waals interactions between CNT and polyethylene hinders vibration leading to crystal-like structure in polyethylene chains. Further, the optimal number of side chains that can be in CNT was found to be three or less. When side chains are three or less, it can suppress the transversal bending of chains and enhance the heat transfer; when it exceeds more than three side chains, phonon scattering dominates and conductivity decreases.

Vega et al. studied the influence of chain branching and molecular weight of polyethylene reinforced with CNT on rheology and crystallization [91]. Ethylene/1-hexene random copolymer, linear polyethylene, and ethylene/1-hexene random copolymers were chosen as matrices. The M_w_ of the matrices and branching were different for three matrices. The matrices were reinforced with CNTs at a w% of 1.04 via *in situ* polymerization via melt blending. It was found that a low percolation threshold was observed for the matrix with lower viscosity. The Newtonian viscosity and steady-state shear recoverable compliance were enhanced due to additions of CNTs in all matrices; however, such enhancements were found to be in linear dependence with M_w_. The matrix with highest molecular weight had a screening effect arising from CNTs network due to high relaxation times involved. The screening effect was due to viscoelasticity interference in measurement of rheological percolation threshold of CNTs. Higher the M_w_ of the matrix, better the nucleation of CNTs. The isothermal and non-isothermal nucleation effects of CNTs increased the molecular weights of the matrix. Stable lamellae formation was found to be inversely related to molecular weight of the matrix. Fractionation quality of the nanocomposite was increased with the branching of the matrix. Shape memory polymers (SMP) is an attractive field of study due to its ability to remember original shape. Depending on the number of temporary shapes, it is classified as double SMP, triple SMP, and so forth. Wang et al. reported triple SMP of polyethylene cross-linked with polycyclooctene (PCO) and CNTs [92]. The specialty about this project was that CNT fillers were dispersed in PCO. The microstructure depends on the volume fraction of each component in it. Up to 30 vol %, PCO was in the dispersed phase and at 50 vol %, it formed a co-continuous matrix with PE. At 90 vol %, PCO was the continuous phase and PE was the dispersed phase. Crosslinking the bicontinuous phase will provide double networks for production of triple SMPs. SEM studies conclude MWCNT fillers were selectively dispersed in PCO phase only for PCO-MWCNT 15 vol %/PE 70/30 vol % nanocomposite. The boundaries between the phases are less visible in phases with MWCNT fillers and electrically conductive networks were affected significantly. Thermal analysis and X-ray diffraction analysis suggest PCO/PE blends are immiscible. Crosslinking of blend and nanocomposite with 2,5-dimethyl-2,5-di(tert-butylperoxy)-hexane (DHBP) initiator T_m_ values decreased and inversely related to the concentration of DHBP. The decrease in Tm was attributed to the hindrance of chemical crosslinking. The triple SMP prepared with nanocomposite exhibited triple shape memory effects (SME) in a dynamic mechanical thermal analyzer and required only 150 V for the SME of PCO phase. Employing fillers in only one phase of the blend decreased a total of the amount of filler to be used in the nanocomposite; additionally, it found to exhibit synergetic enhancement of mechanical properties.

#### 2.3.2. Polystyrene CNT nanocomposites

Polystyrene (PS) nanocomposites prepared with carbonaceous fillers would be of immense help to attain insulation materials with low conductivity by acting as a black body to shield radiation. Polymeric foams are conventional insulation materials to prevent heat loss. Insulation gases used in the past such as chlrofluorocarbon, hydrochlorofluorocarbons to enhance the insulation properties of polymeric foams poses significant hazards to the environment. Super-thermal insulation properties are essential for saving energy costs. Gong et al. synthesized bimodal polystyrene MWCNT nanocomposite (PS/MWCNT) foams using supercritical carbon dioxide by melt blending process [93]. The largest expansion of nanocomposites with appropriate temperature for saturation of CO_2_ and adding pentane to plasticize the matrix. Such prepared nanocomposites had high IR absorption index, and radiative absorption efficiency also increased up to 31 fold with the amount of MWCNT used in the PS matrix. The lowest thermal conductivity achieved for the nanocomposite without any insulation gas 30 mW/m-K. The incorporation of MWCNTs in the PS matrix reduced the radiation and enhanced the optimal expansion ratio which was responsible for such low thermal conductivity. Additionally, the MWCNTs decrease the radiative heat transfer by 8.5 mW/m K. An additional advantage of PS nanocomposites is that the morphology can be easily manipulated by tuning the foaming pressure and temperature. Tuning the morphology gives the flexibility to design nanocomposites that have a strong IR absorption index that effectively prevents both radiation and conduction and lowers the overall thermal conductivity [94]. In another study by Gong et al., PS foam with MWCNT was prepared by supercritical CO_2_ for thermal insulation [95]. Such prepared foams had cell size between 5 and 6 µm and high expansion ratio of 18-fold; the gas conductivity decreased due to Knudsen effect by 1.2 mW/m.K. The radiation with a wavelength smaller than the cell size was attenuated effectively by reflection at the interfaces between cell walls and wavelengths larger than cell size are attenuated by absorption through MWCNTs. Interestingly, the MWCNTs used in the PS matrix decreases the radiative thermal conductivity; however, the solid conductivity increased according to Glicksman model and Rosseland model. At 2 wt % of MWCNT concentration, radiative thermal conductivity decreased by 86% and radiative contribution reduced to 3.5% of the total thermal conductivity. Polymers loaded with ferroelectric materials were investigated as embedded capacitors owing to its flexibility, adhesion strength, and its ability to mold. However, the ferroelectric materials when used in large amounts reduce the advantages mentioned above. Arjmand et al. had investigated CNTs as fillers in the PS matrix, due t its unique properties and suitability of this material in broadband dielectric properties. PS/CNT nanocomposites with different loading of CNTs wt% were prepared by solution mixing technique [96]. At higher CNT loading, insulator–conductor transitions were observed for these materials, when the percolation threshold was met. Beyond the threshold percolation, the conductivity kept increasing and followed by a plateau was reached with increased MWCNT concentration. The plateau signifies the formation of the three-dimensional conductive network. At low MWCNT concentrations, the AC conductivity was increasing with frequency and DC conductivity was very low. When the CNT concentration increased, the DC current catch up with AC conductivity at high frequencies. The real permittivity and imaginary permittivity of nanocomposite at 100 Hz dramatically increased with CNT concentration (5.22 X 10^4^ and 3.28 × 10^7^) than the pristine PS matrix (2.71 and 0.01), respectively. The increase in the value of real permittivity was attributed to a large number of nanocapacitor structures generated in the nanocomposite due to CNT, whereas the imaginary permittivity increase was due to a larger number of dissipating charges, enhancement of conductive network formation, and boosted polarization loss. The AC conductivity was frequency independent in the conductive region and sensitive to the frequency in a linear relationship in the insulative region. The real permittivity decreased with frequency due to charge polarization relaxation, and imaginary permittivity reduction with frequency in the conductive region due to reduced available time for free electrons to sweep the network and interfacial polarization relaxation. PS is considered a versatile polymer as it has features such as good optical transparency, cost-effective, good electric insulation, and mechanical properties. As we know, dispersion of CNT in the polymer matrix is critical to bring the best properties of the nanocomposite; Amr et al. synthesized the PS nanocomposite with CNT functionalized with stearyl alcohol [97]. The stearyl alcohol forms an ester linkage with carboxyl functional groups of CNT. The nanocomposite was prepared by thermal bulk polymerization in the absence of initiator. PS nanocomposite with unfunctionalized CNT and pure PS also prepared to compare the effect of functionalization. The Young's modulus was highest for nanocomposite with functionalized CNT at a loading of 0.1 wt % and 0.5 wt % than the pure PS and PS with unfunctionalized CNT. The increased Young’s modulus was attributed to enhancement in the dispersion of CNT due to stearyl alcohol. The increase in stress transfer was due to better interfacial interactions between polymer and filler. T_g_ was decreased significantly for functional PS/CNT nanocomposite with 0.1 wt % and 1.0 wt % loading as the functionalized CNT scavenges the radicals produced in the polymerization reaction. Storage modulus G’ and loss modulus G’’ were measured for PS/CNT nanocomposite and functionalized PS/CNT nanocomposite at 0.1 and 1 wt %, the measured values were found to increase with increasing concentration; however, for 0.1 wt % functionalized CNT, the values were lower than pure PS, suggesting functionalized CNT behaves as plasticizer. The presence of CNT influenced the properties of nanocomposite at lower frequencies significantly; however, the effect fades out at higher frequencies due to shear thinning. Viscoelastic properties increased with the concentration of filler; however, for low loading of 0.1 wt % for functionalized CNT C-18 do not have any significant increase as the interaction between the filler and matrix was not established. Additionally, the presence of CNT enhanced the thermal properties significantly as indicated by TGA and DTG studies.

Guiliiani et al. fabricated a novel skin temperature sensor based on MWCNTs dispersed in styrene-based polymer poly(vinylbenzyl chloride) derivative with triethylamine (PVBC_Et_3_N) [98]. This PS-based ionomer is known for its effective dispersion of CNTs at high concentrations in polymer matrix via interaction with weak van der Waals and cation–pi interactions. Upon detangling the CNTs by ultrasonication, the ionomers surround the CNTs immediately, thus shielding the strong pi–pi interactions in CNTs that prevent the agglomeration of CNTs. Variation to resistance (94.5–2.6 k Ω) was significant when MWCNT concentration changed from 13.2 wt % to 17.6 wt %; beyond that CNT concentration, the variation was steady and smooth indicative of creation new effective percolation pathways in the polymer matrix. When CNTs have semiconducting or metallic character, they show resistivity that is proportionate to temperature, which makes it useful for nanocomposites for construction of temperature sensors. The resistance variation of nanocomposite found to have a linear relationship with CNT loadings. The nanocomposite with the lowest loading had the highest sensitivity to a negative temperature coefficient of −0.004 K^−1^ which was comparable to metal sensors. Additionally, at MWCNT to polymer ratio of 1:20, the resistance of the sensor recovers to its original value upon the return of nanocomposite to room temperature indicative of the good reproducibility of sensor response after several cycles.

It is well known that the alignment of CNTs in the matrix is crucial to the end-user properties of the nanocomposite. Advancements are being made to produce such nanocomposites; however, the challenges in the characterization of alignment of CNTS and matrix are ominous and complicated due to non-crystallinity of the materials, the elemental identity of the filler, and the hindrance from matrix prevents the use of diffraction and spectroscopic methods. Makarova et al. came up with novel magnetic susceptibility measurements that offer critical insights pertaining to the orientation of CNTs and PS molecules [99]. The ability to make such measurements was by incorporation of iron-based nanoparticles in the nanotube cavity that act as anisotropic magnetic species. PS nanocomposites were prepared with wt % ranging from 1 to 5 via stretching method. PS matrix with CNTs was found to stretch in a uniaxial direction to provide the nanotube alignment, which was confirmed by magnetic susceptibility measurements made in three perpendicular directions. The anisotropy property of the nanocomposite increased by a fold order due to intrinsic susceptibility of CNTs. The strong diamagnetic character of nanocomposite ensued from the graphitic nature of the CNT lattice, where the polymer chains found wrapped to align CNTs. The alignment was at its best in a nanocomposite with smallest CNT concentration. The properties of the polymer matrix are significantly altered when CNT surface is physically or chemically modified. Ionic liquids were employed to modify the surface of CNT, followed by it was used in polymeric nanocomposites. Such prepared nanocomposites improved the tribological properties owing to its effective lubrication. Espejo et al. studied the viscoelastic properties and long-term stability of nanocomposites prepared using ionic liquid ([OMIM] BF4) modified CNTs via dynamic mechanical analysis and timetemperature superposition principle [100]. The glass transition temperature of nanocomposites was not affected by the presence of CNTs at 1 wt % loading. However, the activation energy was increased in nanocomposites for glass transition than the pure PS; particularly, the highest increase was seen in a nanocomposite with MWCNT modified by the ionic liquid. Storage modulus variation with temperature offers clues about the loss of stiffness as temperature increases. The loss in stiffness of nanocomposite can be attributed to poor compatibility between the filler and matrix. The presence of CNTs in PS increases the onset of the storage modulus to longer duration at 313 and 333 K. The highest increase in time for onset of storage modulus was observed for MWCNT modified ionic liquid by a factor of 28. The conclusion from this study was that ionic liquid modified CNT can improve the long-term stability of PS at service temperature conditions. Functionalization of CNTs introduces the dipole–dipole interactions between the CNTs and polymer matrix, which in turn, promotes dispersibility of CNT filler. Khan et al. functionalized the CNT with oleic acid which enables the CNT aqueous dispersion and tethering of reactive functional groups on CNT side walls [101]. The PS/CNT formed through *in situ* emulsion polymerization. The molar mass of polymer and structure of nanocomposite were controlled via functionalization and use of chain transfer agent (CTA). The presence of CNT decreased the molecular weight of PS, and the decrease in molecular weight was inversely related to CNT wt %. Utilization of CTA along with CNT had increased the molecular weight with increase in CNT wt %; however, the overall, molecular weight is significantly lesser than pure PS. Presence of CNT without CTA in the matrix improved the electrical properties of PS at low frequencies; however, at high frequencies, the impedance was gradually plateaued. Presence of CTA along with CNT decreased the impedance further. The electrical properties observed were due to traversing direction of the applied electrical field at low frequencies that were outside the polymer particles. The electrical behavior of nanocomposite was shifted in EIS studies at high frequency due to travel of the electric field through the particle, whereas the travel of electrical field was outside the nanoparticles. Overall enhancement in electrical properties due to CNT and CTA indicate entanglement of CNT in the matrix which was confirmed by TEM studies. The capacities measurements of nanocomposite with CTA were promising for applications in sensors.

Nanocomposites typically exhibit desired EMI values and percolation threshold, at higher filler concentrations only. Maiti et al. have synthesized nanocomposites of polystyrene with MWCNT and graphite nanoplate (GNP) via *in situ* bulk polymerization in the presence of polymerized PS-GNP microbeads [102]. Through this approach, they were able to obtain the nanocomposite with 20.2 dB EMI shielding value at low loading of MWCNT concentration of 2 wt % and GNP loading of 1.5 wt %. At an optimized ratio of PS-GNP-MWCNT, the electrical conductivity was achieved at 9.47 × 10^−3^ S cm^−1^when GNP loading was 0.29 wt % and MWCNT loading of 0.3 wt %. In the nanocomposite matrix, there was random GNP-GNP network formed where MWCNT acts as a bridge which was the key for attaining high electrical conductivity and high EMI shielding value. Field emission scanning electron microscopy and TEM studies suggested the CNT-GNP-CNT network develops the spatial arrangement for pi–pi interaction between the aryl rings in the PS matrix. Polymer films and foams are cost-effective and have weight-affected properties and usefulness of these materials are realized in applications when impact strength, thermals, and electrical insulation and lightweight structures are desired. Dennis et al. had reported fiber-reinforced polymer composite with CNTs prepared at low-temperature calendaring process for the production of foamable thin film plasticized materials [103]. AIBN was used as foaming agent and acetone is to plasticize the nanocomposites. The use of acetone during the synthesis decreases the T_g_ of PS; however, in the presence of CNT, the T_g_ increases. The foam synthesized had cells in the size of microns and thickness of walls in submicrons. The use of CNT had increased the onset of decomposition temperature with the highest onset observed for 12 wt % of CNT. The foaming time is the most critical parameter in the quality of foams produced. Low foaming times produce larger and uniform cell size.

The synthetic strategies to prepare polymer nanocomposites with fine dispersion and interconnected MWCNTs in thermoplastics such as PS are challenging. Oliviera et al. proposed anisotropic self-assembly as a technique to control the formation of long-range interconnected networks of CNTs in PS along with better interfacial compatibility of CNTs to the matrix [104]. This was made possible through grafting MWCNTs with PS on the sidewalls of nanotubes. The homogeneity of the nanocomposite was highly dependent on interfacial interaction between the PS matrix and PS-grafted nanotubes which in turn generate positive mechanical effects (elastic modulus) in the final material. Anisotropic self-assembly of grafted nanotubes demonstrated by them was compatible with extenuating processing and reaction conditions via encapsulation and customized optimization of raw materials. The crack propagation studies and strain upon stress studies indicated lack of interfacial interaction between CNT and PS matrix which is essential for withstanding stress and strain. Chemical grafting of CNT with PS was proved to be advantageous in enhancing the nanocomposite final properties such as electrical conductivity and electromagnetic shielding in an easy fashion. Sachdev et al. prepared PS/MWCNT nanocomposite by dispersing it in PS powder via tumble mixing in the dry state and then pellets were made by hot compression [105]. The DC conductivity of nanocomposite increased abruptly about 12 orders upon addition of 0.05 to 0.1 wt % MWCNTs. The reason for such an increase in conductivity was attributed to the fine dispersion of MWCNTs in the PS matrix due to high impact forces with the stirrer and walls of the tumbler. It was believed that at pellet formation stage, discrete conductive aggregates which grow into continuous conductive paths at percolation threshold. The shielding effectiveness increases slowly at low wt % of MWCNTs and increases faster at higher MWCNT concentration. At higher loadings of filler, conductive mesh network gets closely packed, hence its ability to absorb electromagnetic radiation increases resulting in an increase of shielding effectiveness. The return loss showed an increasing trend at with MWCNT loading except for very low loadings up to 0.1 wt %. The anomaly at such low loadings was due to the interaction of wave with a probability of voids formed during the processing of composites. Shore D hardness decreased with increase in MWCNT loading. At 5 wt %, it had shore D hardness value of 69 suggests strong interfacial bonding between MWCNTs and PS matrix, which makes it ideal to withstand product applications.

Preparation of nanocomposite via *insitu*, anionic and atom transfer radical polymerization requires additional raw materials such as initiator and terminator which is not cost-effective. Solvent blending claims to have easy and good control on the dispersion of CNTs in the polymer matrix even at high loadings of CNT when it utilizes ultrasonication. Sen et al. studied the effect of carboxylated MWCNT (cMWCNT) on structural, thermal, and rheological of the nanocomposites prepared by ultrasonic assisted solution mixing [106]. TEM studies indicated good dispersion at low loading and aggregations found in few regions at higher loading. TGA analysis indicated enhanced thermal stability of nanocomposites which was attributed to nano-confinement and barrier effect of fillers. On average, hardness value of the material increased about 7.5% due to the addition of MWCNT. The storage modulus and loss modulus increased with filler loading and with angular frequency. Convergence of modulus values at a higher frequency indicated liquid to solid transformation of nanocomposite due to the decrease in mobility of polymer chains by geometric confinement of CNTs. The nanocomposite had high elasticity and delayed relaxation due to the presence of CNTs which was effected by filler–filler interaction and matrix–filler interaction. The dispersion of CNT in the polymer matrix is challenging due to the high inherent melt viscosity of the base polymers. Shrivastava et al. reported selective dispersion of MWCNT in a continuous 3D network through the exclusion of some volume of the base matrix [107]. Such exclusion of volume was made by the introduction of high impact polystyrene beads (HIPS) during the polymerization process. Further polymerization was done in presence of polybutadiene rubber. FESEM studies suggested that nanocomposite had a phase with bulk copolymerized HIPS along with the CNT and HIPS beads with a discernible amount of CNT covering surface. The excluded volume created by HIPS beads facilitates the probability of formation of more number of conducting paths in the matrix. The percolation threshold was achieved for the MWCNT wt % of 0.54 in the matrix. Electrical conductivity of a maximum of 1.15 · 10^–5^ S/cm was achieved at 60 wt % of HIPS and 0.6 wt % of MWCNT. These values are relatively higher for similarly prepared nanocomposite by conventional methods such as melt mixing and solution blending. Additionally, the parameters such as bead size, bead adding time, and bead concentration also significantly influence the final properties of the nanocomposite.

The flammability and smoke production are factors that limit the use of PS in applications of construction materials. MWCNTs used in the PS matrix were used as a flame retardant and smoke suppressant in HIPS by Yan et al. [108]. HIPS/MWCNT nanocomposites with various weight fractions of fillers were prepared by melt blending process. Crosslinking of nanotubes was evident around 5 wt % and 10 wt % as found in TEM studies. Presence of MWCNT did not affect the degradation process of HIPS and purely played the role of filler and thermal stability enhancement was attributed to the Labyrinth effect. Smoke production rate, total smoke release, smoke factor, heat release rate, mass loss rate all found to be reduced due to the presence of MWCNT in PS matrix and reduction is proportional to the amount of loading of filler. The reduction in above values was attributed to charring mechanism. The residues of composite found to have a two-layer structure in sub-microscale and micro network structure which has a thin skin layer at the surface of residue and another expanded cellular layer under the skin layer. Presence of those two layers enhances the thickness and volume of residue. Additionally, investigation of both layers indicated the presence of MWCNT interconnected to form a network-like structure which was also responsible for its good insulation behavior to suppress the flammability and smoke production of nanocomposites.

#### 2.3.3. Polyaniline nanocomposites

The discovery and development of conductive polymers by the Nobel Prize winners Alan J. Heeger, Alan G. MacDiarmid, and Hideki Shirakawa in chemistry (2000), has inspired scientists in exploring the excitements in this field. The interest in polyaniline (PANI) research over other conducting polymers was due to its doping characteristics, tunable electrical conductivity, pH-responsive properties, and its stability under ambient conditions. Doping in PANI can be attained via protonic acids or redox reactions. These characteristics of PANI make it very attractive for its utility in the field of sensors.

**Figure 4.**
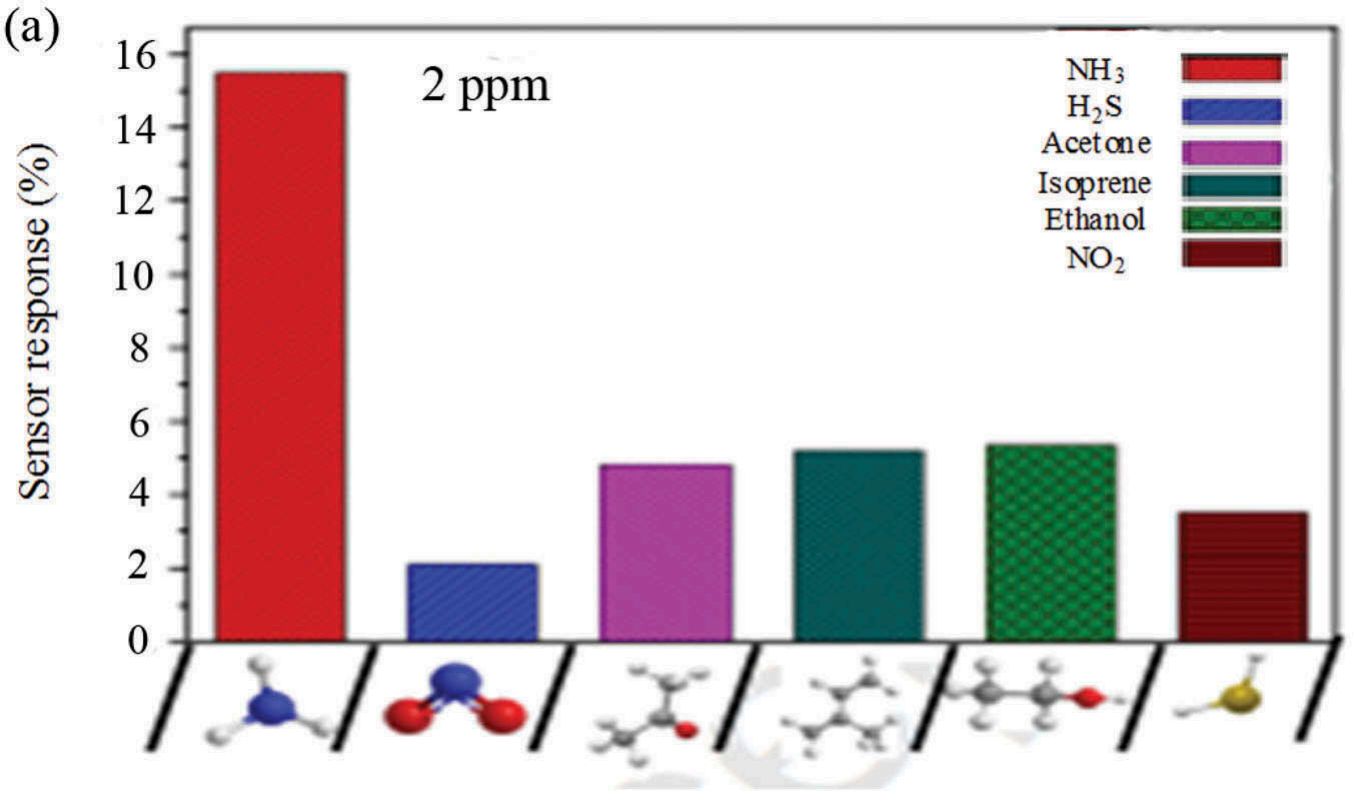
Selectivity sensing studies of PANI/MWCNT toward various gaseous molecules (adapted from reference [109]).

The challenge in designing sensors is based on conductive polymer nanocomposites sensor from failure/breakdown due to polymer degradation, low sensitivity, or incomplete recovery. These shortcomings were addressed by synthesis of PANI-coated MWCNTs that were prepared by *in situ* oxidative polymerization for exclusive ammonia detection (Figure 4) at ambient temperature [109]. Uniform PANI coating as a thin layer offered large surface area and provides several active sites for the adsorption of ammonia gas leading to sense the ammonia as low as 2 PPM. Additionally, the nanocomposite was found to have a very short recovery time of 6 s. The ultrasensitivity and excellent recovery rate were attributed to enhance charge transfer and humidity was found to have negligible interference on sensor response. When the functionalized MWCNT was coated with MWCNT, in contrary to several previous reports, the response time and recovery rate ability of nanocomposites were significantly lower than unfunctionalized MWCNT.

**Figure 5.**
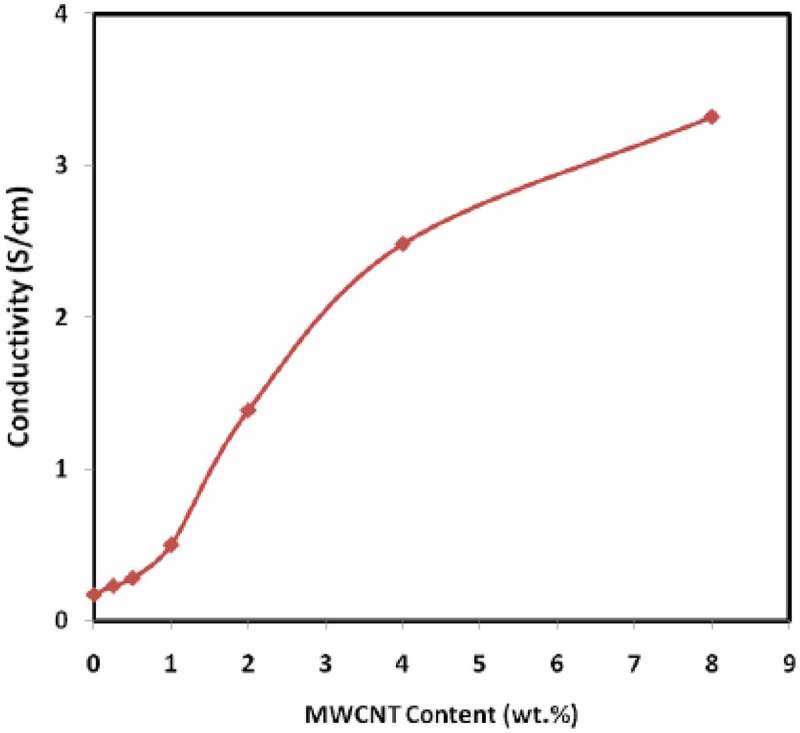
Room temperature electrical conductivity of PANI/MWCNT nanocomposite versus the wt % of MWCNT (adapted from reference [114]).

The cycling stability for PANI supercapacitors is limited due to swelling and shrinking of PANI that could lead to degradation of the electrode during cycling. The degradation is a consequence of volume change due to doping/dedoping. It can be addressed by using composite structures [110–112]. Singu et al. reported the synthesis of PANI/MWCNT nanocomposite with core-shell like structure through *in situ* emulsion polymerization [113]. The specific capacitance of 360 F g^−1^ was observed at a discharge current density of 0.4 A g^−1^ for the nanocomposite. The capacitance retention was 98.3% for over 5000 charge/discharge cycles. The superior performance of the electrode was attributed to the high porosity of the coral-like PANI shell over the MWCNT conductive core. Such core structure enhances the approachability of an electrode to an electrolyte. Self-supported (binder free) PANI/CNT nanocomposite was prepared using high-molecular weight polyethylene oxide (PEO) as carrier polymer through electrospinning process [114]. TEM studies revealed, CNTs were aligned with nanofibers by over 1 μm and had nanofibers with smaller average diameter due to jet elongation achieved by higher charge density imparted by CNT. The smaller diameter of CNTs decreased electrical resistance. The specific capacitance of 385 F g^−1^ at a current density of 0.5 A g^−1^ was found at maximum. Maximum capacitance retention of 81% was obtained at 5 A g^−1^ after 1000 cycles. The superior capacitance retention and specific capacitance values were attributed to inter-fiber porosity in the nanocomposite which facilitates ion transport and diffusion; additionally, the interconnected nanofiber network facilitated incessant electron conductivity. Flexible symmetric and asymmetric supercapacitors were designed with PANI/CNT over carbon cloth [115]. The nanocomposite with CNT functionalized had exhibited high capacitance values regardless of scan rate for symmetric and asymmetric supercapacitors. Between functionalized asymmetric and symmetric PANI/CNT nanocomposite supercapacitors, asymmetric conformation had higher supercapacitance values. The ability of the nanocomposites to use as flexible energy storage devices, electrochemical performance measured at 45° and 90° of symmetric and asymmetrically functionalized PANI/CNT found not affected by the bending angle. The functionalized CNT used in supercapacitors favored the fast penetration of electrolyte that facilitates ion diffusion and thereby decreases the charge transfer distance leading to a superior electrochemical performance in symmetric and asymmetric forms. Further, the carboxylic functional groups improved of capacitors by decreasing the surface resistivity and enhancing the surface wettability. At high current density, the efficiency of charge/discharge cycles was about 90%; meanwhile, at low current densities, symmetric devices performed better than asymmetric. The mechanical and electrochemical corrosion performance of PANI nanocomposite modified with amino functionalized CNTs was studied by Gasem and coworkers [116]. The aminofunctionalized PANI/CNT nanocomposite had exhibited enhanced corrosion performance to 3.5% NaCl solution. The resistance to corrosion was attributed to interfacial interaction of CNT and PANI through polymer/filler bond stability. In another study by Bachdev et al., CNTs functionalized by carboxyl groups were used as filler to prepare PANI nanocomposite via oxidative polymerization [117]. In that preparatory procedure, HCl was used as dopant and ammonium persulfate was as an oxidant. Such prepared nanocomposites were investigated for electrical conductivity studies. Up to 1% nanofiller concentration, the enhancement in DC conductivity was minimal, but conductivity enhancement rapidly increased beyond 1 wt % and reached the conductivity of 3.32 S/cm at 8 wt % (Figure 5) which is 20 times more than pristine PANI. The enhancement was due to synergism between PANI and CNT forming an interpenetrating 3D conductive network.

**Figure 6.**
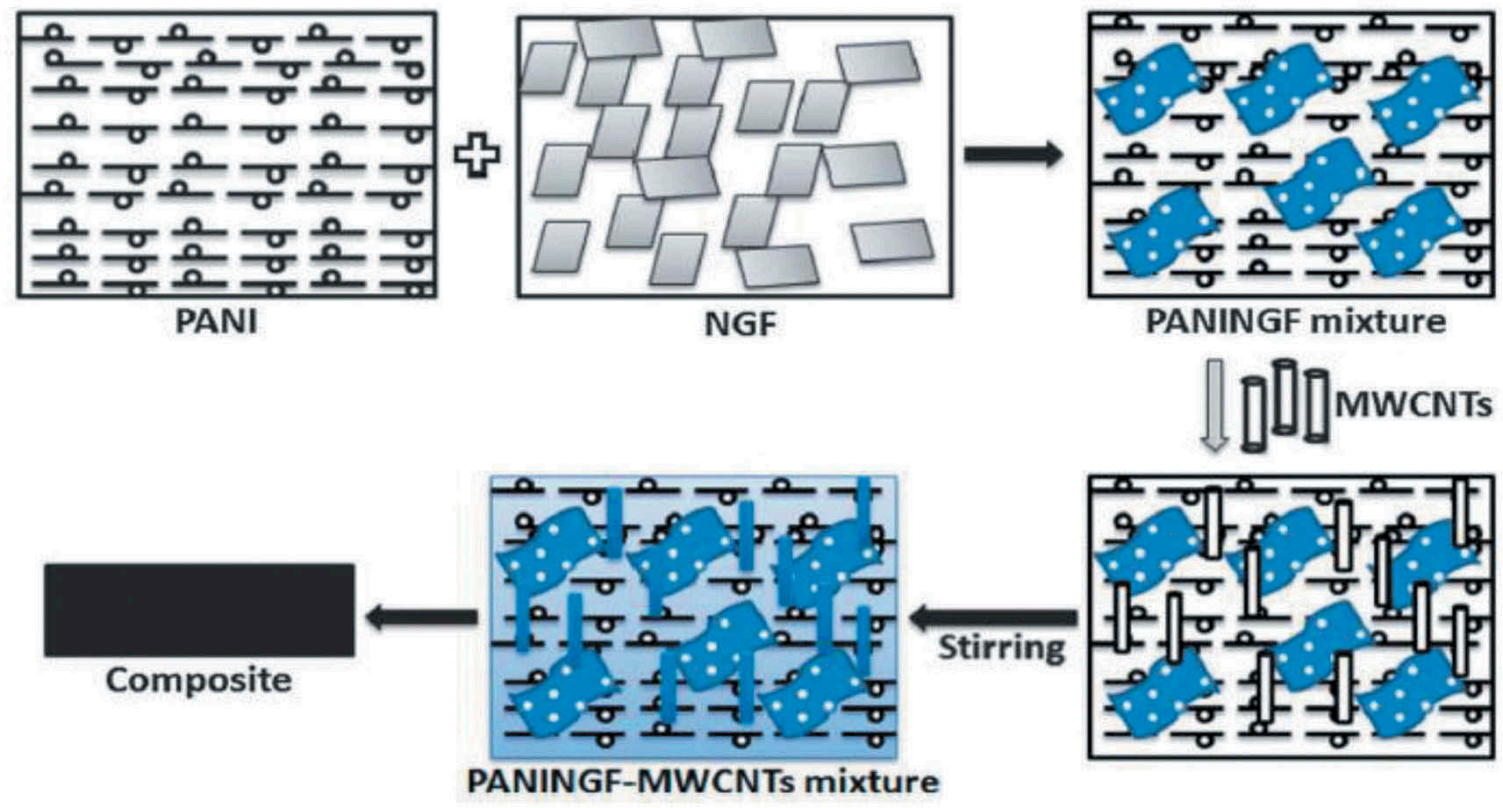
Schematic for preparation of PANINGF-MWCNT (adapted from reference [118]).

Role of oxidants such as Cr(VI) and ammonium persulfate in the synthesis of PANI/CNT nanocomposites were studied by Huang et al. [118]. The nanocomposites were prepared by surface-initiated polymerization. A nanocomposite prepared with Cr(VI) oxidant had negative permittivity, while the oxidant ammonium persulfate had exhibited positive permittivity. Like permittivity, negative giant magnetoresistance of −2% was observed for Cr(VI) and positive giant magnetoresistance of 2.4% was observed for ammonium persulfate oxidant. The switching effects of giant magnetoresistance for nanocomposites with different oxidants were explained by the differences in calculated localization length by wave-function shrinkage model and magnetoconductivity theory. Gupta et al. developed PANI nanocomposite (Figure 6) with dual reinforcement components (natural graphite flakes, MWCNT) that are in micro and nanophase with improved electromagnetic interference shielding effectiveness (EMI-SE) [119]. The natural graphite flakes (NGF) was ball milled for several hours to produce *in situ* graphene. The amounts of MWCNT in the nanocomposite increased the shore hardness and electrical conductivity. At 10 wt %, the shore hardness was 91 and EMI-SE was about 98 dB. The enhancement of EMI-SE was due to the synergetic effect of functionalized MWCNTs and *in situ* generated graphene and oxidative polymerized PANI via increasing the surface charge polarization and reduction in carrier mobility. The dielectric constant and dielectric loss increased with increase in MWCNT concentration due to the difference in electrical conductivity values of MWCNT between PANI.

**Figure 7.**
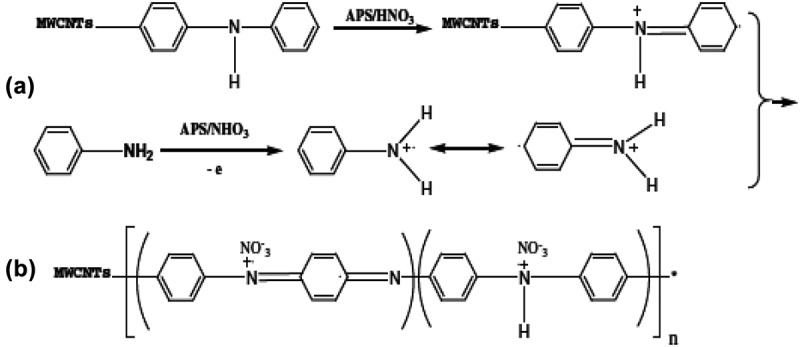
Scheme for preparation of core/shell PANI/MWCNT. (a) Cation radical polymerization and (b) polymerization on the surface of MWCNTs (adapted from reference [119]).

Core/shell protuberance-free PANI/MWCNT were prepared via interfacial chemistry of aryldiazonium salts [120]. On that attempt, MWCNTs were covalently functionalized using diphenylamine groups; upon functionalization, PANI was grafted on the surface of functionalized MWCNT through *in situ* oxidative polymerization of aniline. The MWCNT forms the core and PANI form the shell. The conductivity measured using four probe method for core/shell nanocomposite was as twice as unfunctionalized PANI/MWCNT nanocomposite. Diphenylamine used for surface modification served as a useful matrix for charge transport, increasing the effective charge delocalization was attributed for increased conductance of core/shell nanocomposite as illustrated in Figure 7.

PANI/MWCNT nanocomposite doped with titania and ruthenium oxide was explored as a film on the gold electrode for epinephrine sensing application in the presence of interference molecules [121]. The electron transport properties towards oxidation of epinephrine were found to be best when electrodes were coated with nanocomposite doped with titania or ruthenium oxide than over the PANI/MWCNT nanocomposite. The tafel values for titania-doped PANI/MWCNT and ruthenium oxide-doped PANI/MWCNT are 0.448 and 0.443 V/decade, respectively. The limits for the electrodes doped with titania and ruthenium oxide were 0.16 and 0.18 µm, respectively. Further, the differential pulse voltammetry studies indicated clear peak separation between epinephrine and interference amino acid molecules and ascorbic acid. The electrochemical impedance spectroscopy had electron transfer resistance of 3.27 and 5.2 kΩ for titania and ruthenium oxide metals, respectively. The electrodes with metals doped exhibited good stability for the analyte and its ability to re-use was very good even after saved in the refrigerator for 3 days and reasonable resistance for electrode poisoning. In a study by Zhang et al. PANI/MWCNT nanocomposites were prepared by a green fabricating method known as cryogenic grinding without the use of dispersant [122]. Upon cryogenic grinding, it was consolidated by spark plasma sintering method (SPS) method. Presence of MWCNT in the nanocomposite increased the electrical conductivity to 1.59 × 10^2^S/m which was significantly larger than pure PANI. The enhancement was due to strong pi–pi interaction between the pi-bonded surface of the CNTs and conjugated PANI. No evidence for a change in Seebeck coefficient value was observed due to the incorporation of MWCNT. Maximum power factor was measured to be 10.73 × 10^−8^W/mK^2^ at 30 wt % of MWCNT and 380 K; additionally, it had low thermal conductivity values. The thermoelectric properties measured for the cryogenic grinding method is better than nanocomposites prepared under *in situ* polymerization due to enhanced dispersion achieved via truncation.

PANI-based conducting polymers can be synthesized by inexpensive ways, and they are stable under ambient conditions. Additionally, they possess wide potential range as an ionic electronic conductor. Bavio et al. synthesized PANI nanostructures and PANI-CNT nanostructures using CNTs that are functionalized and non-functionalized. The non-functionalized CNTs are MWCNT and functionalized CNTs are MWCNTs subjected to oxidizing pre-treatment. The functionalized MWCNT produced PANI nanotubes of approximately 15 µm length, with an outer diameter of approximately 95 nm. However, non-functionalized MWCNT yielded composites of nanoparticles of 90 nm in diameter. Voltamperometric runs of PANI-CNT nanostructures had better capacitive behavior as the nanocomposite allows large surface area and better access to the electrolyte in the internal and external porous structure, and by supplying faradaic pseudo-capacitance generated on the superficial oxygenated groups developed during the acid pretreatment. Through internal resistance, the galvanostatic charge/discharge measurements of PAN-CNT functionalized nanocomposite had a highest specific capacitance value of 1744 F/g at 2 A/g. The superior performance PANI-CNT functionalized nanocomposite stems from strong coupling between CNT and polymer chains via charge transfer process between quinoid rings of PANI and CNT [123].

In a study by Chang et al., multiscale structured preparation of PANI and its nanocomposites with MWCNT was reported using nanocasting technique for fabrication of high roughness surfaces using a biomimetic template (Xanthosoma sagittifolium) and evaluated their capacitive properties. Hierarchical structures in the biological world are responsible for functional integration. With that motivation, PANI-3D film nanocomposites analysis by SEM indicated successful replication of leaf-like multiscale morphology from the leaves on the surface of the electrode. The electrochemical capacitive performance of PANI nanocomposite film coincides well with surface morphology of electrode. The high roughness of electrode surface generates reaction sites for redox reactions and facilitates rapid diffusion of ions. Specific capacitance as high as 535 F/G at a current density of 1 A/g was observed for 5 wt% loading of MWCNT in PANI. Further, the PANI-MWCNT nanocomposite exhibited lowest ohmic drop, with MWCNT playing the role of percolator that has high electrical conductivity and interconnectivity. Further, the PANI-3D composites synthesized using biomimetic template method do not require additional binder materials [124].

Poly-pyrrole is one of the conducting polymers that was extensively investigated owing to superior physicochemical properties. Cui et al. developed poly-pyrrole nanocomposite with lactate oxidase as a catalyst with and without CNT. This was the first work to be reported for the biocatalytic synthesis of poly-pyrrole. The negatively charged lactate oxidase enzyme molecules served as seeds to positively charged oligomers of poly-pyrrole under synthetic conditions. Thus, the enzyme played a dual role – seed and catalyst. Lactate oxidase serves as seeds for the formation of poly-pyrrole lactate oxidase composite; however, CNT took the role of seeds in the poly-pyrrole lactate oxidase CNT nanocomposite. The reaction time, composition of components in nanocomposite determines the thickness and morphology of the poly-pyrrole lactate oxidase CNT nanocomposite. The tunable nanoscale structure, morphology of nanocomposite provides opportunities to tailor the nanocomposites for important applications in electronic and biosensor devices. The nanocomposite with CNT had more ohmic-like property, indicative of enhanced conductivity which finds themselves in an application where electron transfer is desired. The I-V curves of nanocomposite with CNT had ‘S’ shape reflective of semiconductor-like characteristics [125].

**Figure 8.**
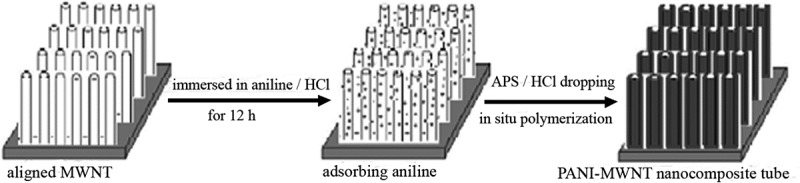
Scheme explaining the preparatory procedure of well-aligned PANI nanocomposite tube film.

PANI is extensively investigated conducting polymer owing to its relative ease in synthesis, electrical conductivity, and its stability to environmental conditions. These properties can be further enhanced by the addition of MWCNT. Feng et al. reported one such PANI nanocomposite tube films that were well aligned with MWCNTs as shown in Figure 8:

The synthesis of PANI nanocomposite tube films was achieved via *in situ* polymerization. PANI changes from fiber-like texture to granular form upon the nanocomposite tube formation. The diameters of nanocomposite tube range from 10 to 25 nm and thickness of PANI on MWCNT increased with time. The MWCNTs were found to be trapped in PANI chains, which was confirmed by IR studies. In-plane C-C bond deformation suggests interactions between quinoid ring and nanotubes, which was facilitated by *in situ* polymerization. Such interactions favor the charge-transfer process leading to enhancement of transport properties of the nanocomposite. The room temperature conductivity of the nanocomposite tube increased by magnitude than PANI, and the temperature dependence of the resistivity observed to be weaker than PANI due to greater charge carrier delocalization. The observed enhancement in physical properties in nanocomposite tubes did not affect its chemical properties [126].

Electrically conductive hybrid epoxy adhesives filled with p-toluenesulfonic acid doped with PANI and CNTs were prepared by Khandelwal et al. At 0.1% of CNT, the epoxy-PANI hybrid nanocomposite, the electrical conductivity increased by seven order, due to the interconnection of PANI particles by CNT bridges enabling 3D conductive network. The epoxy resins in hybrid nanocomposites prevent the molecular motion and dampen diffusion of reactants, thus facilitates the crosslinking of components. CNTs act as an interlocking agent by positioning itself between epoxy and PANI which was evident by increased Tg temperature. The presence of CNT in the hybrid nanocomposite decreased the shear strength due to the generation of linear solid to solid contact points. SEM micrographs of hybrid nanocomposites were indicative of homogeneous dispersion of components of the composite [127].

Li et al. reported high surface area PANI-CNT nanocomposite foam paper that had 3D networked with uniform submicrometer pores. Ni/CNT composite was first generated as a thin sheet structure on sinter-lock Ni-microfibrous structure through catalytic chemical vapor deposition. PANI-sol coating was conducted on CNTs implanted on Ni-microfibrous structure as this allows free-standing PANI that was uniform and dense shell of PANI-Ni-CNT nanocomposite with a unique core-shell structure. The 3D network of the nanocomposite serves as electrolyte reservoir which will help reduce ion-transfer distance from bulk electrolyte to nanocomposite surface. The self-support/free-standing nature of nanocomposite does not require additional binders for molding and permits to use them in supercapacitor electrodes directly. The electrochemical impedance measurements of hybrid nanocomposite exhibited a small semicircle was observed indicative of low interfacial charge transfer resistance due to Ni serving as a current collector and CNT acts as nanoconducting wire to link the charge storage PANI film. The specific capacitance of nanocomposite was measured to be 725 F/g at 0.5 A/g and exhibited a high energy density of 22 W h kg-1 at 2000 W kg-1 based on total electrode mass. Further, the galvanostatic charge-discharge testing of nanocomposite at 5 A/g, the specific capacitance can be retained after 300 cycles and lasted over 1000 cycles indicative of good stability [128].

CNT/PANI nanocomposite film that was transparent and free-standing was synthesized via interfacial (water–toluene interface) polymerization was reported by Salvatierra et al. The synthesis and processing were achieved as a one-pot reaction. The polymerization found to begin at the surface of CNT, which is reflective of the fact that CNT acts as seeds for heterogeneous nucleation. At higher concentrations of CNT, polymer growth was around the CNT, however, at higher concentration monomer, homogeneous nucleation of polymer nanofiber aggregates formed. Formation of PANI fibers can be achieved only when the interfacial polymerization was conducted without stirring. Stirring the reaction mixture prevents formation of freestanding films. The polyaniline chains in the nanocomposite found to be in planar conformation and had more polaronic structure. The π-π interactions between the polymer and CNT tend to stabilize by stacking over polymer chains over CNTs. (CNT)^δ-^…(PANI)^2+^

(HSO4)^−2^, interactions makes the transfer of part of electron density from PANI to CNTs possible. Pickering emulsions were attributed to the stability of the interface that leads to the formation of free-standing films [129].

#### 2.3.4. Polylactide (PLA) nanocomposite

**Figure 9.**
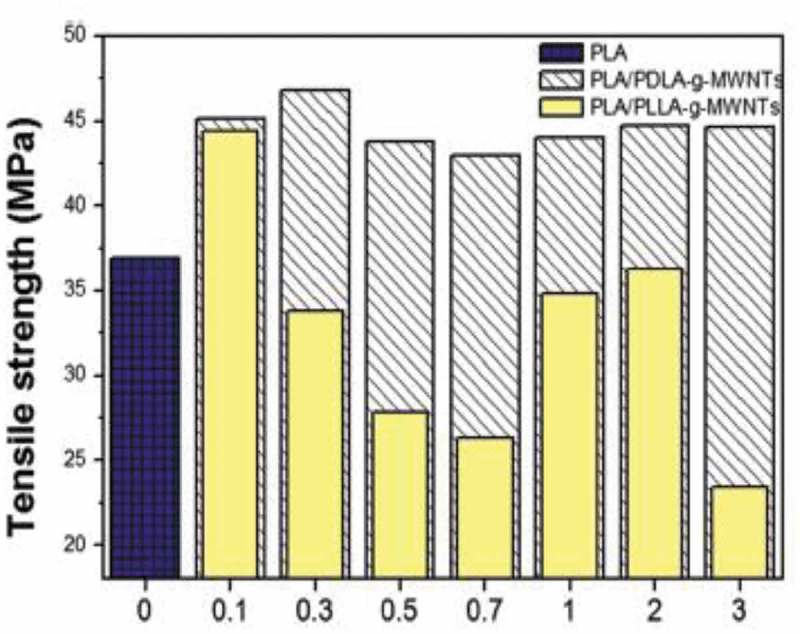
Tensile strengths of neat PLA (blue) PLA nanocomposite with MWNTs modified with PDLA (gray) and PLA nanocomposite with MWNTs modified with PLLA (yellow) (adapted from reference [141]).

Biodegradable polymers have myriad applications in areas of packaging, biomedical, agriculture to name a few owing to its excellent mechanical properties, cost-effectiveness, biocompatibility, and easy processing conditions. PLAs are superior to conventionally known polymers derived from petroleum products as it consumes lesser energy and releases lesser carbon dioxide as investigated by life cycle assessment [130–133]. The attraction of PLA exists in its economic and environmental considerations. Lactides are dimers of lactic acid, which undergoes ring opening polymerization to form polylactide. The common source of lactic acid is corn starch. Lactic acid has one chiral center, due to that it exists two configurations, D, L. The polymer of lactic acid with exclusive isomers of D and L are known as PDLA and PLLA, respectively. The relative proportions of isomers found to influence the properties of the final polymer. The positive attributes of PLAs are its stiffness and strength; however, the disadvantages are brittleness, low elongation at break, slow crystallization rate, poor heat resistance, which limit its applications and usefulness in large-scale industrial applications [134–138]. CNTs role as filler in PLAs was able to overcome several disadvantages mentioned above and put the nanocomposite in a better perspective [139]. PLA synthesized exclusively with L-isomer typically known as PLLA. High crystallinity in PLLA (hPLLA) has a high melting point, which serves as an efficient nucleating agent. The presence of hPLLA as a nucleating agent in PLLA/CNT nanocomposites enhances the thermomechanical properties and heat distortion properties and facilitates the formation of conductive networks via volume exclusion effect [140]. Effects of different crystalline morphologies and crystallinity of PLLA were investigated by Zhang et al. toward electrical conductivities and mechanical properties. Conductive percolate threshold decreased by 60% at 1 wt % of hPLLA in PLLA/CNT nanocomposite. Additionally, the electrical conductivity increased by 7 orders of magnitude under optimal hPLLA and MWCNT loading along with a significant increase in Young’s modulus and elongation at break of nanocomposites. PLA polymers reinforced with carbon nanofillers effects interfacial adhesion and homogeneous dispersion. Yang et al. used MWCNTs grafted with PDLA as reinforcing fillers and its effect in the PLA matrix were studied [141]. Use of PDLA promoted nucleation and increased overall crystallization rate of the polymer matrix. The tensile strength of PLA nanocomposite was fairly constant over various concentrations of modified MWNTs with PDLA, and it is significantly higher than pure PLA. Similar studies with MWNTs modified with L isomer were inconsistent with trends (Figure 9). The interaction between PLA and MWNTs modified with PDLA was very strong regardless of concentration, however, for MWNTs modified with PLLA exhibited aggregation and resulting in poor interfacial interaction.

**Figure 10.**
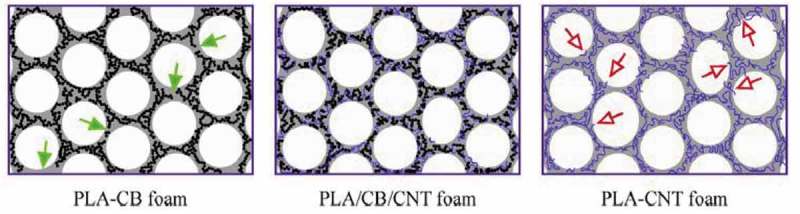
Schematics of carbonaceous filler networks in the PLA foams. The solid curves and the black dots represent CNTs and CB, respectively. The solid arrows indicate broken or invalid pathways of the CB networks, and the hollow arrows ‘dead’ ends or branches of the CNTs located at the broken cell wall, which have no contribution to the conductive networks (adapted from reference [142]).

The inclusion of carbonaceous materials in PLA serves as a reinforcer and imparts electrical conductivity. Wu et al. prepared PLA nanocomposite with carbon black (CB), CNT individually and another nanocomposite that contains both of them together in the PLA [142]. PLA nanocomposite containing individual carbonaceous filler was observed as loosely entangled components. However, when both CB and CNT added in the PLA matrix and foamed with supercritical CO_2_, a synergistic relationship between the matrix and PLA is observed as in Figure 10. In the ternary phase, CB restricts the CNTs movement during the synthesis which facilitates penetration probability of their free ends into the cell wall. Consequently, the cells with unbroken wall structure formations aid setup of conductive filler networks which in turn enhance the conductivity of the nanocomposite. In another study by Wang et al. similar enhancement in mechanical and electrical properties of PLA/CNT nanocomposite prepared by the extrusion process was enhanced due to the incorporation of CNT [143]. In this study too, CNT improved the crystallinity of nanocomposite by playing the role of the nucleating agent. Tensile strength increased up to 33.12%. Further, the nanocomposites synthesized by this procedure exhibited distribution and dispersion of CNTs in PLA matrix which was evident through scanning electron microscopy studies.

**Figure 11.**
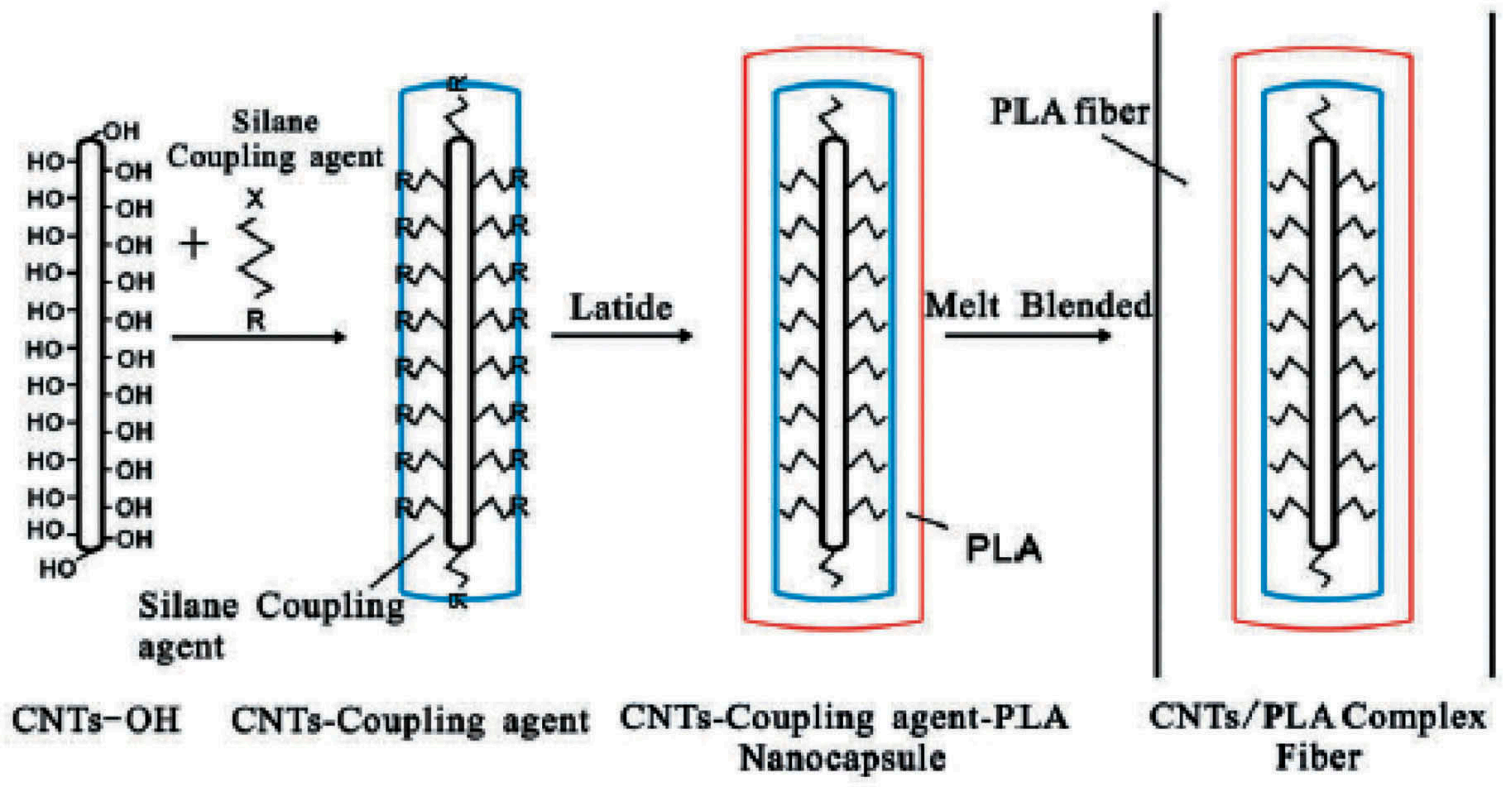
Schematic representation for the formation of core-shell PLA nanocomposite fiber (adapted from reference [145]).

Liu et al. attempted to overcome the brittleness of PLA by preparing PLA/CNT nanocomposite with core-shell structure [144]. The nanocomposite had a hydroxylated-CNT kernel which was grafted with flexible macromolecular siloxane chain to serve as the coupling agent. The grafted siloxane and CNT kernel were sealed by a PLA shell to form a nanocapsule to prevent the coupling agent being destroyed during processing of nanocomposite. Additionally, the PLA seal prevents adhesion of coupling agent chains. The PLA matrix was generated on this shell (Figure 11). Through this preparatory procedure, when CNT nanocapsules are less than 1 wt%, it had homogeneous dispersion. The CNT nanocapsules increased the crystallinity of nanocomposite significantly by acting as nucleating agents. Additionally, the crystallinity improved the tensile strength of the nanocomposite by 30.62% and elongation of nanocomposite fiber by 32.2%. The findings of this study are very relevant to use PLA fiber nanocomposite in applications such as garments, agricultural protecting nets were toughness matters.

**Figure 12.**
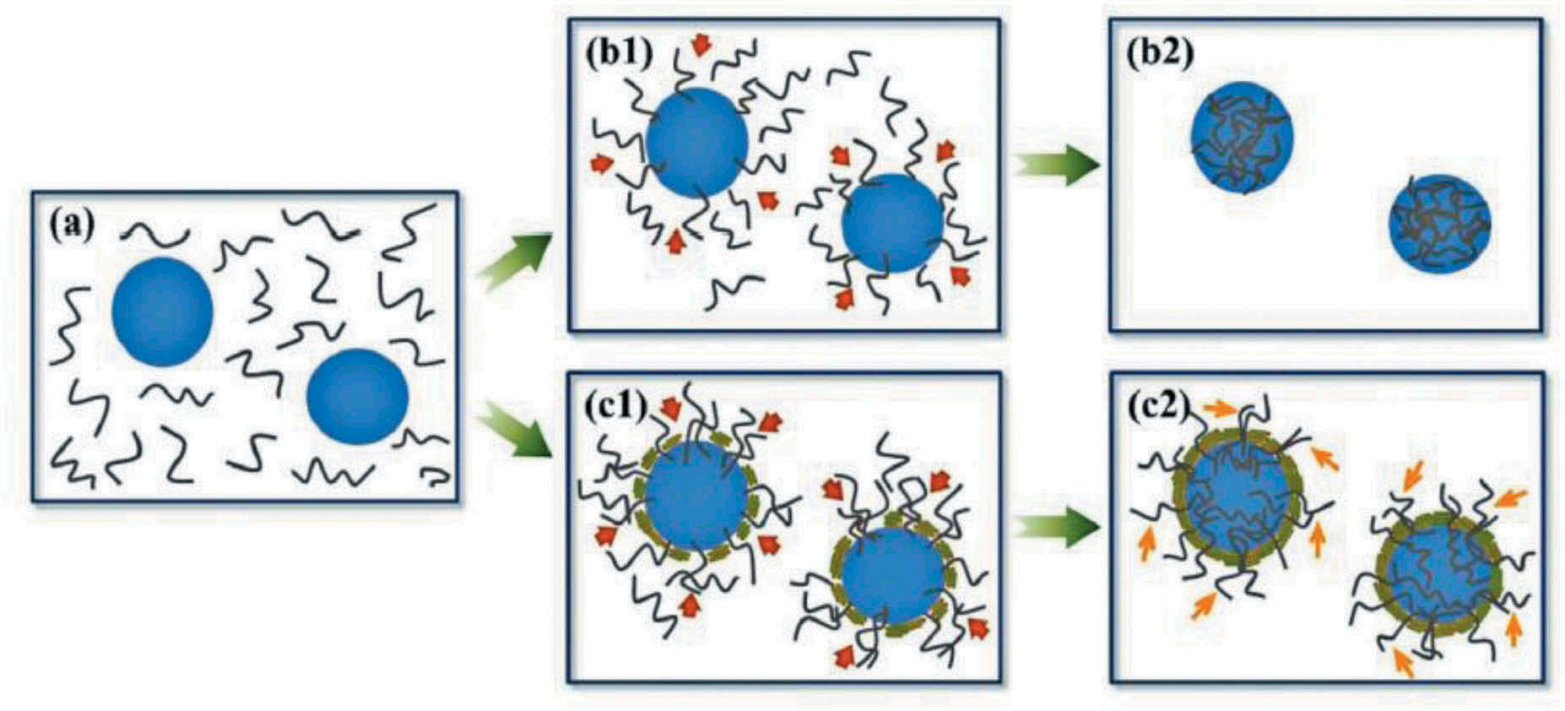
Schematic representations showing the transfer and localization of MWCNTs in PLLA/elastomer blends with a sea-island phase structure during melt-mixing process: (a) MWCNTs are pre-dispersed in poor wetting PLLA matrix and subsequently melt-mixed with a more favorable EGD elastomer phase; (b1-b2) generally, MWCNTs prefer to transfer from PLLA matrix to dispersed elastomer particles; (c1-c2) if SC crystallites could be rapidly formed at the interface of PLLA/EGD blend, some MWCNTs would be anchored by the interface-localized SC crystallites when they transfer across the interface (adapted from reference [145]).

Planting nanoparticles at the interface of immiscible polymer blends enhance the properties of the blend and add functionalities to the nanocomposite. The polymer blends of PLLA with appropriate elastomer are known to toughen the PLLA. However, the thermodynamical immiscibility of the blend leads to poor interfacial adhesion which poorly transfers stress from the PLLA matrix. This was addressed by Lu et al. by anchoring high aspect ratio MWCNTs in the interface of PLLA and grafted ethylene acrylic ester copolymer (EGD) via interface localized stereocomplex crystallites as anchoring agents (Figure 12) [145]. The nanocomposites were prepared by melt blending process. MWCNTs pre-dispersed in poor wetting PLLA matrix favors transfer to more favorable EGD phase and stereochemical crystallites forms rapidly at blend interface. The EGD phase plays a dual role of physical barriers and nucleating agents to induce stereochemical complex crystallization. Such prepared nanocomposites had found to have remarkable interfacial strength and low percolation threshold.

## 3. Graphene nanosheets

Graphene is an exciting material that has pulled in much consideration in the last years and is being widely investigated in view of its properties, which have been characterized with so many superlatives. In this review section, we aim to present an overview of the progression of research in graphene application in polymer composites which has resulted in enhanced electrical, mechanical and thermal properties. The different features of graphene such as properties, production methods, and also models of graphene nanocomposite will be discussed. Hence, this review will be helpful to both learner and professionals.

### 3.1. Background

**Figure 13.**
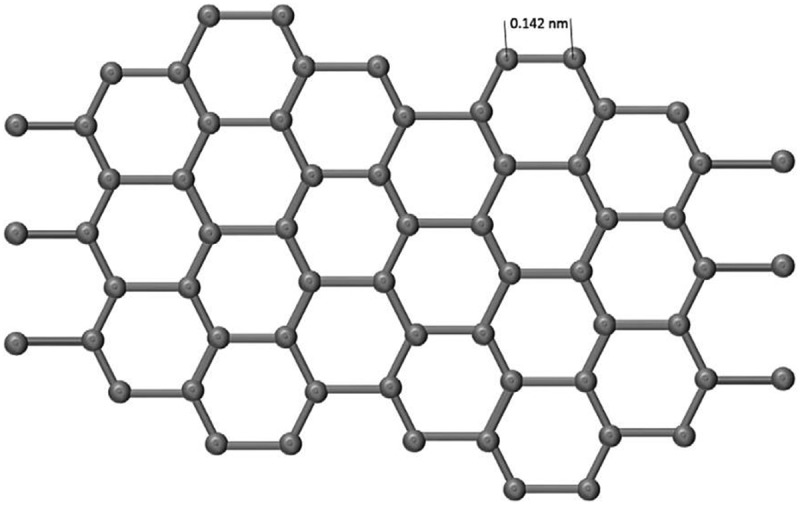
Carbon atoms arranged in a hexagonal lattice, showing C-C bond length of 0.142 nm in graphene structure (adapted from reference [156]).

Graphene is defined as a one-atom-thick planar sheet composed of sp^2^-bonded carbon atoms densely arranged in a crystal honeycomb lattice and is the thinnest recognized material in the world. These carbon atoms are bonded together at a length of 0.142 nm (as shown in Figure 13). It has attracted significant attention from both academic and industries in the investigation of graphene properties, production methods and potential applications [146–148] to be used as a promising electronic material. The main reason behind that is due to its unique Physical properties such as great current density, chemical inertness, high thermal conductivity, ballistic transport, optical transmittance, and excellent hydrophobicity at a scale of nanometer [149,150]. These extraordinary features of graphene mark it as exciting nanofiller in the field of polymer nanocomposites. In addition, graphene has exposed a great application in electronics, photo-catalysis, sensors, medicine and solar cells [151–155]. In recent years, graphene has used as the greatest favorable nanofiller due to resultant brilliant properties, which has opened a new field of polymeric nanomaterials. Importantly, the properties of polymer-graphene composites based on many factors such as (i) the dispersion of graphene layers or fillers, in general, within the polymer, (ii) the interactions of the filler–polymer matrix at the interface, (iii) proportion of the graphene filler to polymer matrix, and (iv) the brilliance of polymer matrix and graphene filler. The important point should be considered is that these features can be different from the methods and fabrication processes [156]. Compared with other carbon filler-based polymer nanocomposites, graphene at very low percolation thresholds allows for huge variations in the electrical and mechanical properties of the composites due to the ultrahigh aspect ratio of graphene [156,157]. This section briefly highlight few models were graphene has been utilized to enhance the properties of polymer composites. In addition to that, we are going to take a quick look at the methods of how graphene and polymer are combined to yield composites.

### 3.2. Fabrication methods of graphene/polymer nanocomposites

The dispersion of the nanofillers is a critical step in polymer nanocomposites for achieving a well-dispersed and homogeneous system of the studying nanocomposite. A well-dispersed state guarantees a maximized reinforced surface area, which will influence the neighboring polymer chains and, thus, the properties of the entire matrix. In this manner, substantial endeavors have been focused on accomplishing a homogeneous and very much scattered framework by growing either covalent or non-covalent functionalization of the filler surface. Most three methodologies that are generally used for incorporation of graphene into polymer matrices are (1) *in situ* polymerization, (2) melt intercalation, and (3) solution mixing is the three main. Importantly, the mechanism for the interaction between graphene and polymers depends on hydrophobicity, molar mass, polarity, reactivity, etc., which should consider picking the fabrication methods [158] as discussed below.

#### 3.2.1. *In situ* polymerization

In this method, graphene fillers and a liquid monomer of interest are first mixed in the presence of a catalyst in some cases [159–162]. Appropriate weigh (0.1 g) of graphene of polymer/G is heated in 1 ml of hydrazine monohydrate at 95°C for 12 h. After this step, filtration is used to collect the resulted composites. To remove extra hydrazine, the mixture is washed many times with DI water and ethanol. The resulted composite has been dried in a vacuum oven at 75°C for overnight. The suggested mechanism of composite synthesis is illustrated in. As a result, this strategy is one of the best ways for polymerization which is started whichever by heat or radiation [163]. A range of polymer nanocomposites has been fabricated utilized this technique such as graphene/epoxy [163] composites which is a good example for utilizing this method, graphene/polyaniline (PANI) composite [164] as a flexible electrode, and graphene/ polystyrene (PS) [165]. The main benefit of this approach is summarized on two points: first, it forms a strong interaction between the fillers units and the polymer matrix, which leads to facilitate stress transfer resulting in a rapid formation of a homogeneous dispersion of the filler in the polymer matrix. Secondly, it provides brilliant miscibility for high filler loading in polymer matrices. On the other hand, this strategy is commonly accompanied by increasing the viscosity of the blend resulting in a restriction of the operation and loading fraction. In addition to that, In-situ polymerization method occurs in the presence of solvents and therefore removing the solvent is another critical problem. Hence, this strategy increases the necessity for additional purification steps to remove the applied solvent [166].

#### 3.2.2. Melt intercalation technique

Melt intercalation method uses a high level of temperature to soften the polymeric matrix and shear force for the nanoparticles to be dispersed easily within the polymeric matrix [167–170]. To best result, the polymer and GNs should stay some days in an oven before mixing at 708°C and then mixed for 10 min. at high temperature with 100 rpm speed. In other words, this polymerization method takes place when the polymer matrix is melted first, and then compounded with the intercalated nanofillers such as graphene which is mixed with the polymer which is melted in the molten state under absolute of inert gas, e.g., N_2_, indicating that no solvent is mandatory in this method, so no need for purification step. This method has found extensive application for preparation of thermoplastic composites [171]. Mainly, a thermoplastic polymer is mixed mechanically with the graphene or modified graphene at the elevated temperatures resulting in a homogeneous mixing of graphitic materials and polymers. There is a wide range of graphene/polymer composites have been prepared by using the application of this method, including polyethylene terephthalate (PET)/graphene nanocomposites [158], polystyrene/graphite nanosheet composites [172], exfoliated graphite/polypropylene nanocomposites [171], etc. The main downsides of this technique are that (i) in the presence of higher amounts of the nanocomposite the preparation becomes difficult, due to the increscent of the viscosity, (ii) the distribution of the nanocomposite fillers in the polymer matrix are relatively poor especially when compared with other methods, and (iii) the possibility of nanoparticles aggregation during the mixing step, due to use heavy shear forces which induces more defects and damage graphene sheets [173].

#### 3.2.3. Solution mixing

This technique is the most fabrication strategy commonly used for nanocomposite polymer compared to all of the production methods discussed above due to its simplicity [174–176] (allows synthesis of nanocomposite polymers with low polarity or sometimes with no polar solvents), the possibility for wide-scale application, and it does not entail particular tool and equipment [156,177]. The Solution intercalation method is based on three steps: (1) diffusion in an appropriate solvent by ultra-sonication or other technique, (2) addition of the polymer, and (3) removal of the solvent by evaporation or distillation. In specific, the polymer is solubilized in a proper solvent and, then, graphene is allowed to swell. Graphene can be distributed without difficulty in a carefully selected solvent, such as chloroform, water, tetrahydrofuran (THF), acetone, dimethyl formamide (DMF) or toluene, outstanding on the poor interaction allowing layers to stack together one by one. At that point, the polymer adsorbs onto the delaminated layers, and at what time the dissolvable is dissipated, the layers reassemble again, sandwiching the polymer to shape the nanocomposites [177,178]. However, it has been reported some challenging problems in polymer solution related to the solubility and dispersity of graphene. They found that most organic solvents are strongly adsorbed on the materials even after careful removal and drying procedure [179] leading to a limitation in solvent blending procedures on graphene chemistry. Therefore, graphene sheets functionalized is strongly needed to avoid against aggregation by increasing the solubility of graphene in corresponding solvents. Some composites have been fabricated using this strategy including poly(methyl methacrylate)(PMMA)/graphene [180], graphite oxide nanoplatelet/poly-urethane (PU) composite [181], and graphite oxide nanoplatelets (GONPs)/polycaprolactone (PCL) [182].

### 3.3. Polymer nanocomposites reinforced graphene

#### 3.3.1. Graphene/epoxy composites

**Figure 14.**
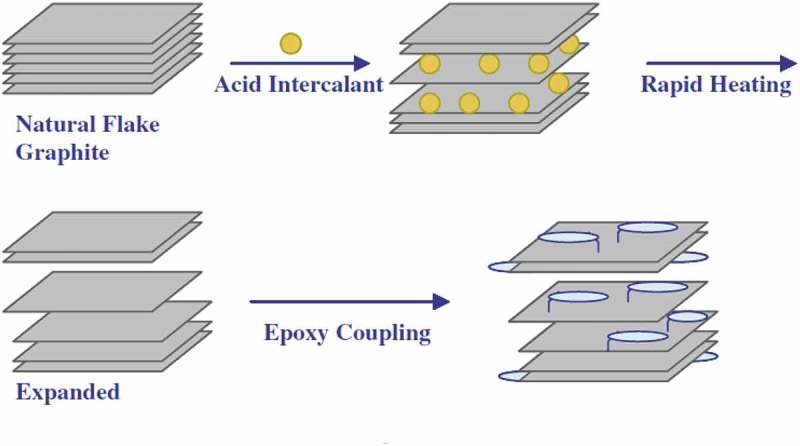
Schematic of graphite expansion and functionalization process (adapted from reference [185]).

Epoxy based material is defined as a thermosetting polymer which extensively used in structural applications due to its convenient heat resistance and mechanical properties. Since their discovery in 1936, epoxy composites have found in applications which require enhanced strength and electrical performance ranging from aerospace, adhesive, electronics, and coating fields. One way to improve the performance is by addition of conductive reinforcing fillers to the epoxy composites such as graphene knowing for its unique mechanical properties [183,184]. In 2010, Millar et al. [185] presented a new approach to graphite nanoparticles functionalization. A schematic of this method is shown in Figure 14.

**Figure 15.**
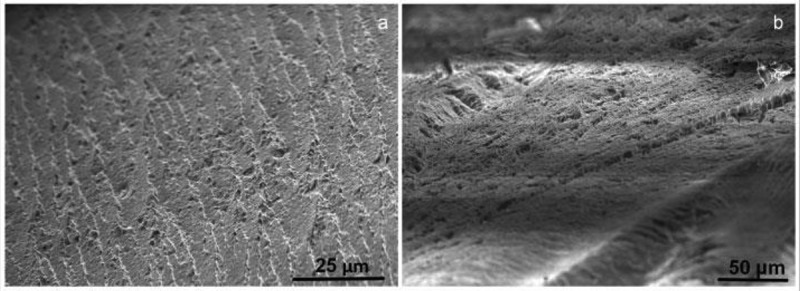
SEM images of (a) 0.13 vol % graphene/epoxy composite (scale bar: 25 µm) produced by solution processing method, and (b) 0.23 vol% graphene/epoxy composite (scale bar: 50 µm) prepared by freeze drying technique (adapted from reference [183]).

Their technique showed minimal disruptions on the sp^2^ graphene sheet with improved material properties. The nano-filler after only 15 min of stirring was finely dispersed with the epoxy resin, and importantly the dispersion process did not exhibit any increase in epoxy viscosity, which benefits the development of this material. The result of the functionalization improved a five order of magnitude in electrical conductivity and a50% and 30% enhancement in stiffness and strength, respectively. Wajid et al. [183] used freeze-dry/mixing and solution processing to fabricate graphene/epoxy composites. The resulted composites displayed greater thermal stability and enhanced electrical and mechanical properties. The well-dispersion quality of the graphene in the epoxy polymer was confirmed by microscopy and XRD. Figure 15 shows a clear comparison between the two methods which demonstrates that the superior dispersion excellence of graphene is accomplished by the solution processing technique. At only 0.46 vol% graphene loading, the modulus and strength improved by 37% and 38%, respectively. In addition, the epoxy composites display a 10–40% growth in the mechanical properties, and also the electrical conductivity was improved by seven orders of magnitude.

**Figure 16.**
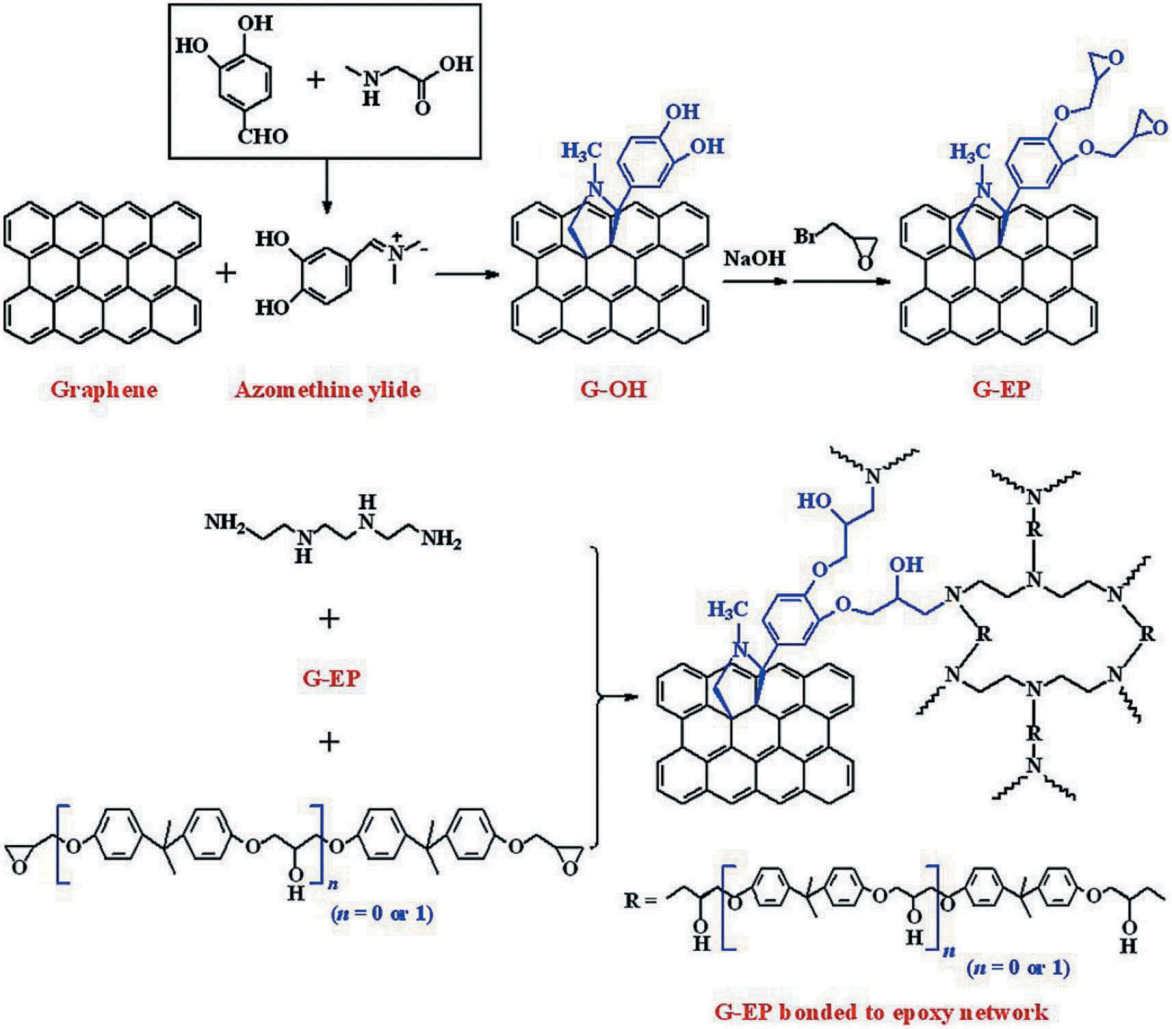
The synthesis of epoxide-functionalized graphene and its application in the epoxy composite (adapted from reference [187]).

In 2014, it has been reported [186] the consequence of graphene oxide (GO) on the mechanical properties of the epoxy composite which was fabricated through solution mixing technique. It was stated that incorporation of only 0.1 wt% of GO improved the mechanical properties of the epoxy by nearly 50%. Moreover, the elastic modulus improved by 35% from the neat epoxy with only 0.5 wt% graphene oxide loading. Recently, Zhao et al. [187] investigated the properties of epoxy composite filled with epoxide-functionalized graphene (G-EP) (see Figure 16 for synthesis method). The Young’s modulus and tensile strength increased by 96% and 116%, respectively; compared to the polymer at only 1 wt% loading of G-EP. The percolation threshold at only 0.33 wt% loading was reached, and electrical conductivity improved from about 1^−17^ to 1^−2^ S cm^−1^ at nearly 2 vol% loading. In addition, thermal conductivity was enhanced by 189% at 10 wt% G-EP loading.

#### 3.3.2. Polystyrene (PS)/graphene nanocomposites

**Figure 17.**
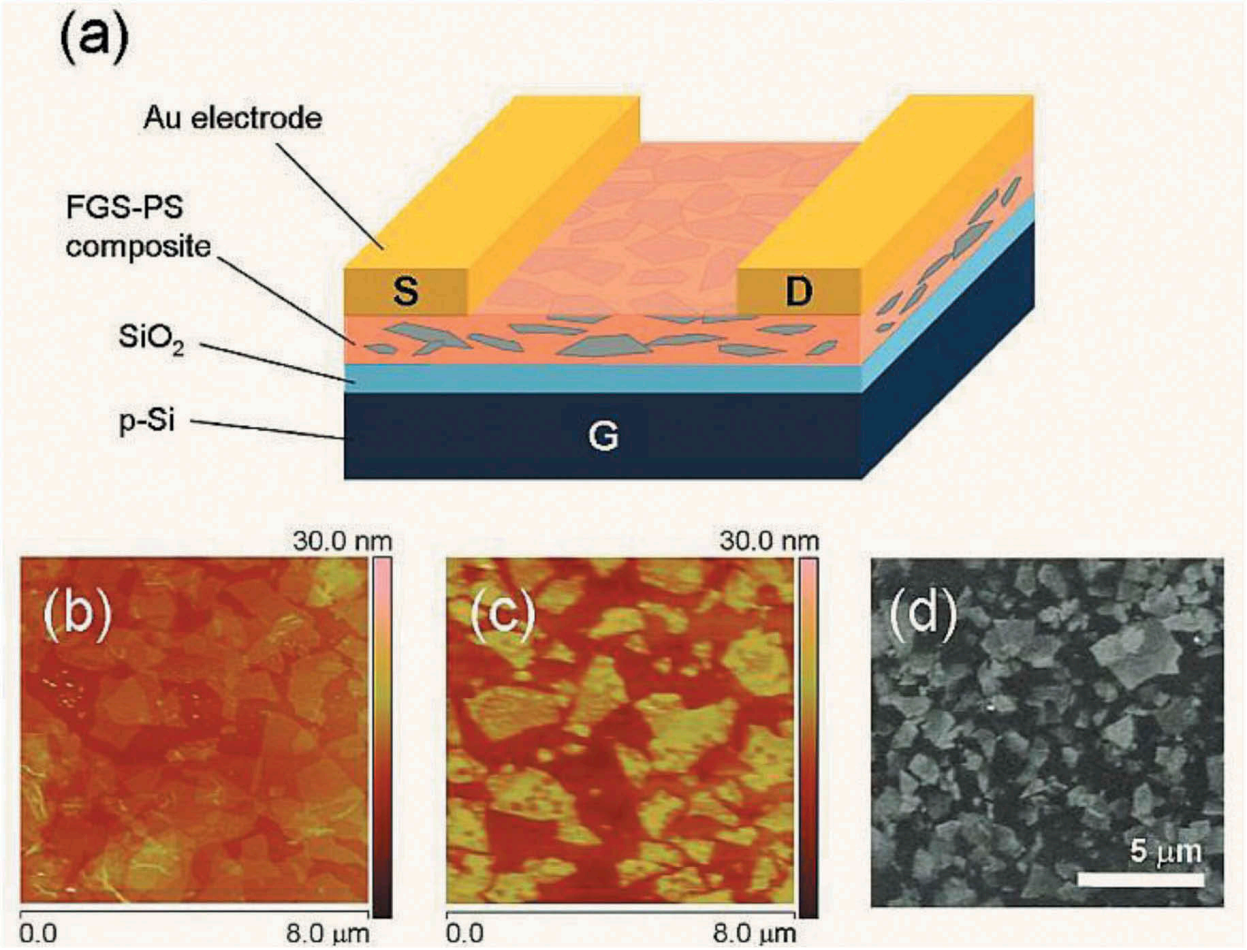
(a) Thin film of FGS-PS composite diagram. AFM of (b) phenyl-isocyanate treated GO and (c) thin film of FGS-PS composite. (d) SEM of typical FGS-PS composite thin film as-deposited. Contrast can be realized between conductive FGS (light) and insulating PS (dark) (adapted from reference [188]).

Polystyrene/graphene composites one of the most nanocomposites have been comprehensively studied for numerous application. In 2009, the electrical properties of functionalized graphene sheets (FGS) which act as the filler and polystyrene (PS) working as the host material had been investigated (PS/FGS) [188] by using a solution blending method (see Figure 17). The results show that the highest mobility values have been achieved by using devices fabricated utilizing the most significant size of FGS yield [188].

**Figure 18.**
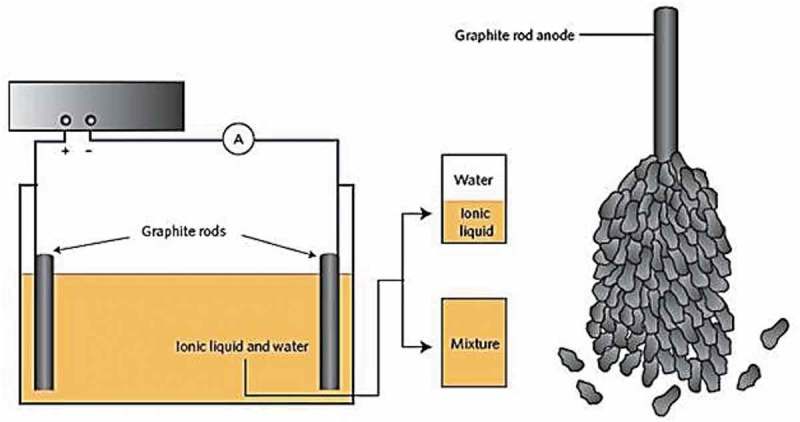
Set-up diagram experimental (left) and the graphite anode exfoliation (right) (adapted from reference [165]).

Liu et al. [165] reported a mild, one-step electrochemical methodology to prepare ionic-liquid- treated graphite sheets through the support of an ionic liquid and water as illustrated in Figure 18. They found that these ionic-liquid-functionalized graphite sheets can be exfoliated into functionalized graphene nanosheets that can be spread homogeneously into polar aprotic solvents molecules, and also no need to further deoxidization. Importantly, the properties of the graphene nanosheets can be influenced by diverse ionic liquids types and also by changed percentages of the ionic liquid to water. There results show that, for room temperature electrical conductivity graphene nanosheet/polystyrene composites produced by a liquid-phase blend route display a percolation threshold of 0.1 vol %, as well as, at only 4.19 vol %; this nanosheet/polystyrene composite shows a high conductivity of 13.84 S m^−1^, that is upper by 3–15 times compared to that of single-walled carbon nanotubes polystyrene composites. Also, they studied the thermal stability of composite and pure polystyrene displaying the degradation temperature of the PS/GNP composite was nearly 50°C greater than that of pure PS. This finding indicates that this improvement is due to the high interaction at the interface of GNP and the polymer matrix, leading to a decrease of polymer chain mobility near the interface and, accordingly, the increase in thermal stability.

Recently, Qi-qi Bai et al. [189] introduced PS/graphene platelets (GNP) composites via one-step process (solution compounding, PS/GNP-S) and two-step process (solution compounding and subsequent melt compounding, PS/GNP-SM). The microstructures and dispersion states of GNPs in the composites were reasonably explored. Compared with the PS/GNP-SM composites, the findings showed that high ability to form the percolated GNP network although the PS/GNP-S composites demonstrated comparatively poor dispersion of GNP particles. In addition, PS/GNP-S samples exhibited high glass transition, low volume resistivity, and mostly improved thermal conductivity.

#### 3.3.3. Graphene/polyaniline (PANI) composites

Using *in situ* anodic electropolymerization (AEP), Wang et al. [164] prepared a graphene/PANI composite paper (GPCP). This composite, interestingly, was exhibited a stable large electrochemical gravimetric and volumetric capacitance of 233 F g^−1^ and 135 F cm^−3^, respectively, and shown a tensile strength of 12.6 MPa. Also, the mechanical strength was improved by 43%.

**Figure 19.**
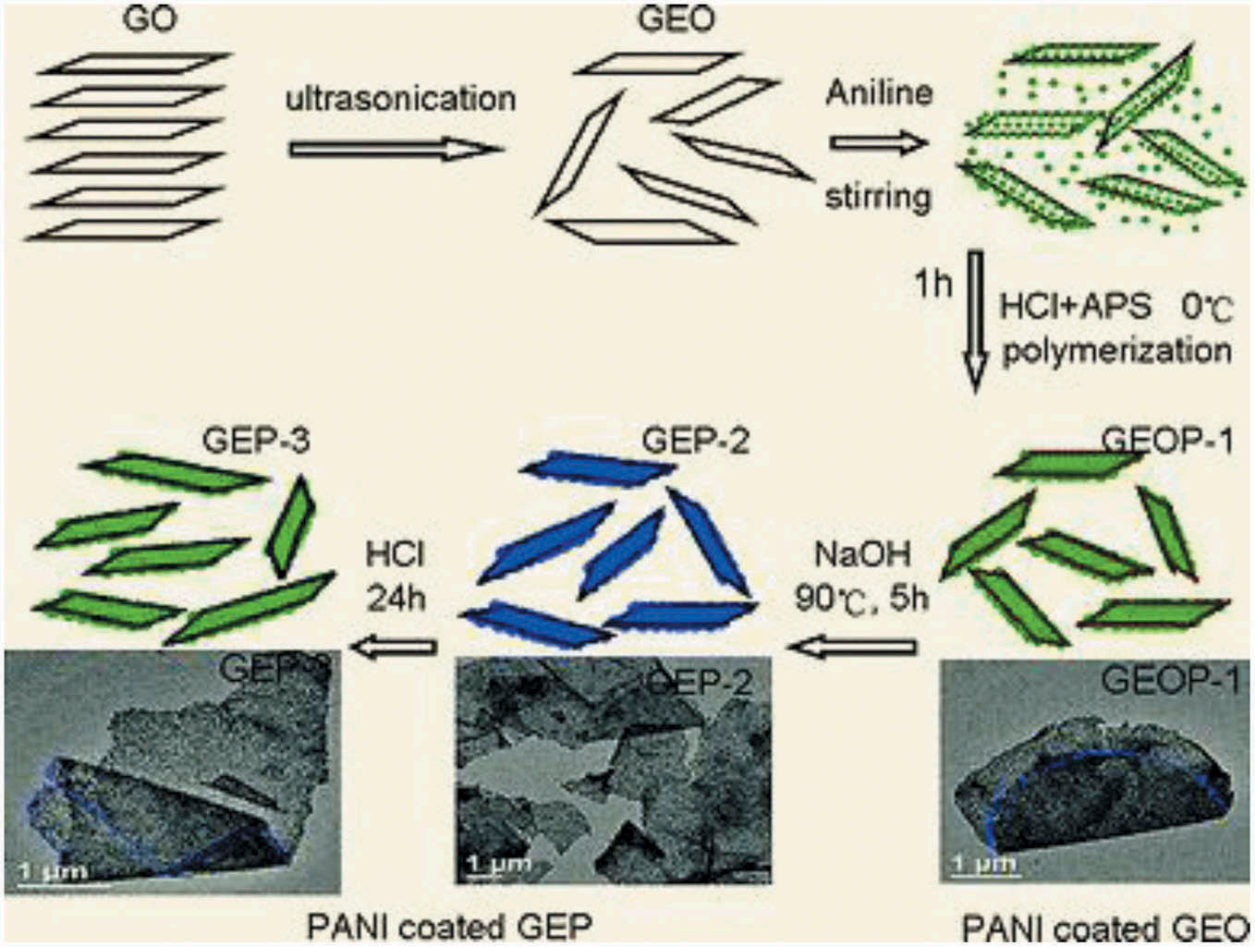
A scheme showing the GEP hybrid materials preparation process of (adapted from reference [190]).

A supercapacitor electrode graphene/polyaniline hybrid material was produced [190] by an *in situ* polymerization-reduction/dedoping – redoping process. To achieve the reduced graphene oxide/polyaniline hybrid material, this product, first, was equipped in an ethylene glycol medium, after that polyaniline in the graphene/polyaniline composite treated with a dedoping reagent such as hot sodium hydroxide solution as illustrated in Figure 19. Afterward, the flexible conducting composites were attained with unaffected morphology. X-ray photoelectron spectroscopy technique and Raman study were used to characterize the chemical structure of the materials under investigation. Compared to pure individual components, the composite material showed: (1) better electrochemical performances, (2) a high specific capacitance of 1126 F g^−1^ which was achieved with a retention life of 84% only after 1000 cycles for supercapacitors, and (3) an enhancement in the energy density and power density.

#### 3.3.4. PVA/graphene nanocomposites

Zhao et al. [191] described the preparation of nanocomposites established on thoroughly exfoliated graphene nanosheets and poly(vinyl alcohol) (PVA). They obtained through a facial aqueous solution. This study shows that (1) a necessary improvement of mechanical properties of the composites at low loading of graphene, (2) a 150% development of tensile strength by only 1.8 vol % loading of graphene. Although great recent nanoscale fillers as carbon nanotubes growth, the required of strong and cost-efficient multifunctional nanocomposite materials has strongly needed. In fact, the difficulties facing researchers are to accomplish a well-dispersion and a high level of interfacial interaction between the nanofiller and the polymer matrix at low filling. Based on that, Liang et al. [192] reported the remarkable method to prepare poly(vinyl alcohol) (PVA) nanocomposites with graphene oxide (GO) by a simple way of water solution processing. They found effective load transfer between the nanofiller graphene and PVA matrix. Also, the molecule-level dispersion and mechanical properties of the nanocomposite are successfully developed. Knowing that adding of only 0.7 wt% of GO resulted in a 76% increase in tensile strength and a 62% development of Young’s modulus.

### 3.4. Outlook of graphene/polymer nanocomposites

**Figure 20.**
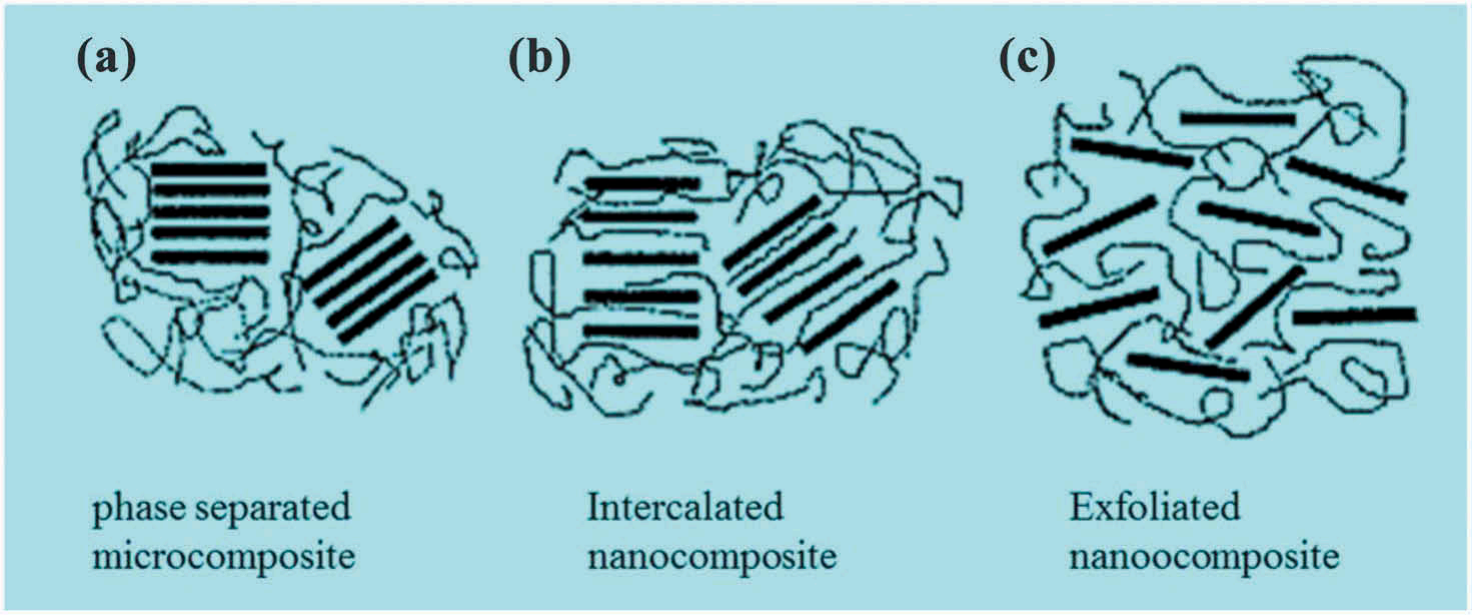
Schematic display three morphological states that are possible with graphene-based nanocomposites: (a) phase separated, (b) intercalated, (c) exfoliated (adapted from reference [196]).

Apparently, graphene shows a lot of features as filler making it promising materials to improve the polymer properties such as mechanical, electrical and thermal properties. Nevertheless, to grasp the full potential of the resultant composites with adequate possessions for broader applications, many obstacles must be overcome. Importantly, the composites properties governed by the well interaction between graphene and the polymer matrix besides homogenous dispersion of graphene in the polymer matrix which enhance the identical load transfer through the polymer matrix. One way to improve the dispersion capability of graphene is by introducing graphene functionalization. Reduced graphene oxide (rGO) or GO are good examples for functionalized polymer utilized as fillers in polymer composites. The main reason behind that is GO contains oxygen functional groups such as hydroxyl, epoxy and carboxylic, which can easily interact with the polymer matrix. Also, rGO compound which produced from the reduction of GO oxide still comprises many of these remaining functional groups leading to increase the spacing of interlayer and reduce the van der Waals force interaction effects between graphene layers [193–196]. When graphene materials are used as nanofillers in polymer-based composites, there are three possible types of morphological phases: (1) stacked, (2) intercalated, and (3) exfoliated as illustrated in (Figure 20). In brief, when the polymer is incapable to interpolate between the graphene sheets, a phase separated composite is made that contains large stacked agglomerates of graphene sheets. More significant features can be achieved if there is a strong interfacial bonding interaction between graphene and the host polymer matrix. While in the exfoliated Phase, exfoliated graphene fragments show a high tendency of interfacial contact with the polymer matrix enhancing the mechanical properties of the composites.

## 4. Fullerene

### 4.1. Background

Fullerene is the third allotrope of carbon after graphite and diamond; and can exist in many shapes as a hollow sphere, tube, and few other forms. First of its series, Buckminsterfullerenes C_60_ (buckyballs) were discovered in 1985 [197] and have the shape of a football. As graphite, the structure of fullerene consists of stacked layers of graphene or of linked hexagonal rings. Carbon in fullerene has sp2 hybridization and possess a tendency to act as electron deficient alkenes and react with rich molecules. An Obstacle in the use of fullerene and its derivatives is their solubility. Fullerenes are sparingly soluble in most solvents. Common solvents of fullerenes are carbon disulfide (CS2)m benzene and toluene. Nevertheless, fullerene is the only known allotrope of carbon which is soluble at room temperature.

### 4.2. Polymer nanocomposites-based fullerenes

**Figure 21.**
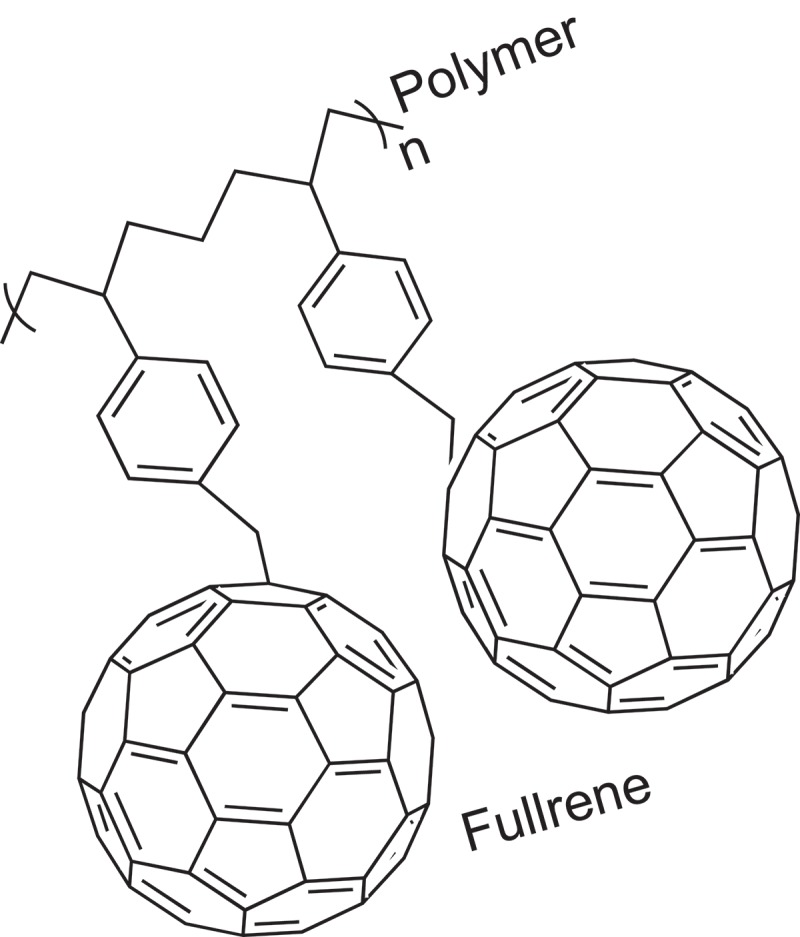
Schematic illustration of polymer-based fullerene as a course of carbon-based materials.

Since their early discovery, fullerenes have been used heavily in nanocarbon material. Of many of its fascinating properties, fullerenes have a spherical π-conjugated structure with large pyramidalization angle which eases chemical functionalize than CNT [198] and therefore causing much higher solubility in many organic and aqueous solvents. One of the most important applications of polymer/fullerenes is carbon fiber-reinforced composites [199], which going to be the main focus of this section (see Figure 21).

The fictionalization of the polymer has many advantages as it permits the preparation of nanocomposites material and improves transmittance in the visible light range. This is mainly due to the diminishing of the π-conjugation by the substation of groups on the fullerenyl C=C double bond moiety. Recently, a synthetic method was developed to give highly polyhydroxylated fullerene C_60_(OH)_36_ having an unusual light yellowish color and high water solubility [200]. Original fullerene C_60_ and polyhydroxylated-fullerenes, C_60_(OH)_12_ and C_60_(OH)_36_, were prepared by Saotome et al. as novel nanocomposite films of polycarbonate (PC) with fullerene derivatives [201]. The addition of hydroxylated fullerenes was studied and showed no significant effect in the visible light transmittance of the films in the range of 400–800 nm, as was shown by ultraviolet-visible spectroscopy. This interesting minimal influence on overall transparency can be explicated by a combined effect of decreased π-conjugation and accumulations of the nanofillers for the PC nanocomposites. Two thermal techniques were applied: differential scanning calorimetry (DSC) and thermos gravimetric analysis (TGA) measurements. Both techniques showed an increase in the thermal stability of PC/C_60_(OH)_12_ film as compared to original PC film. This phenomenon was proposed to arise from the firm polymer interphase area formed around C_60_(OH)_12_ due to the likely hydrophobic interaction in the solution and hydrogen bonding. On the other hand, the lower thermal stability of PC-C_60_(OH)_36_ was observed and assumed to be produced by the partial hydrolysis of the polycarbonate material and the huge accumulation of the C_60_(OH)_36_ particles. A decrease in elongation at breaks and yield tensile strength was observed in the study as showed by the tensile testing of the composites. The particle accumulations might be causing these observations as they act as the introduction points for cracks [200].

**Figure 22.**
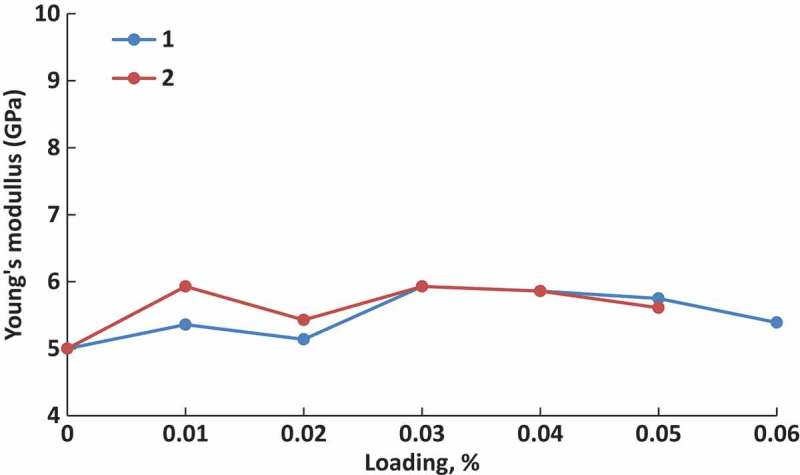
Effect of pure fullerene C60 (1) and of fullerenes C60/C70 mixture (2) content on acoustic Young’s modulus of epoxy resin [201].

In another domain, the mechanical properties of reinforcing polymer nanocomposites based on reactoplasts (epoxy resins) and thermoplasts (polyamide-12) with fulleroids which are C60, a mixture of C60/C70 and fulleroids dust and two different carbon fillers [202]. Polyamine is the catalyst for epoxy resin curing and fullerene C60 is light material and quickly react with an amine group in polyamide. This leads to an increase of local density of amino in curing agent. Therefore, the obtained materials are brittle because of the local high cross-link density. In Figure 22 are given mechanical and acoustic properties of epoxy resins loading with fullerenes. It was concluded in the study that the fillers did not affect the properties of epoxy resins and therefore, the tensile modulus and tensile strength of polyamide-12-based polymer nanocomposites are improved by about 30–40% [201].

The effect of fullerene dispersion on the properties of reinforced epoxy carbon fiber composites was studied by Ogasawara et al. of many mechanical properties were examined: tension, compression, open-hole compression, compression after impact and interlaminar fracture toughness were of interest. Notably, increments of the tension and compression strength by 2–12% were obtained upon the addition of 0.5% fullerene into the matrix resin. Also, the interlaminar fracture toughness of the composite was improved by 60% after the addition of fullerene [203]. A method to identify the properties of the polymer matrix is the nano-indentation method [204,205]. The idea behind this method depends on deforming the material’s surface in a certain load with an indenter. Nano-indentation method were used to determine the mechanical properties of blend films of poly(3-hexylthiophene) (P3HT)/ [6]- phenyl-C61-butyricacidmethylester (PCBM) [206]. Nano-indentation method was studied and reported on nanocomposite of fullerene (C60) and epoxy in order to understand their mechanical properties [207]. Many mechanical properties were enhanced such as: fracture energy, fracture toughness, and ultimate tensile strength by loading of several weight fractions of fullerene in the fullerene epoxy matrix. The morphological effects of loading fullerene C60 into the P3HT and PCBM blend films were reported by Richards et al. and showed [208] that the incorporation of C60 altered the growth rate and morphology of PCBM crystallites. The addition of C60 showed a strong effect on the growth of bulky PCBM crystallites. Decreasing in the fullerene aggregation and crystallite size was observed as a consequence of the addition of fullerene [206].

**Figure 23.**
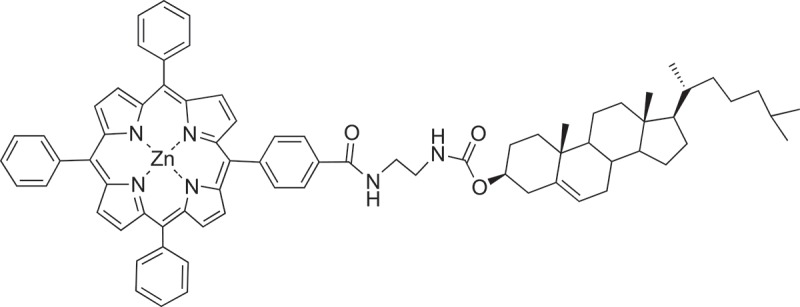
Gelator molecule 1.

The solution route was used to synthesize a fullerene enforced polyethylene oxide nanocomposites. High power sonication was used to ensure dispersion of fullerene in the solution of the polymer. The influence of fullerene nanoparticles on the polymer chain was inspected and revealed an entirely different behavior of C60 dispersed within the polymeric network than using other nanofiller such as carbon nanofibers or nanotubes. This observed phenomenon is due to the low aspect ratio of fullerene in comparison to nanotubes and by the low thermal conductivity of fullerene paralleled to the thermal conductivity of others carbon type nanofiller [209]. Fullerene and its functionalized analogs are widely explored in the fabrication of gel linkages. Supramolecular polyfullerenes [210] were of special interest as a guest (additives) to the gel-nanocomposites [211]. Doping fullerene into the self-supporting gels introduces many advantages since the electrical and optical properties of fullerene are transferred into the newly formed composite [212]. Numerous nanocomposites were synthesized from molecular gelators, some of those small gelators were linked to the fullerene moiety through covalent bonds. Mainly, fullerene composites were made in the gel in the organic medium due to its inherent hydrophobicity. Shinkai et al. reported fullerene-based gel composite formed from organogelator with porphyrin moiety. First, the gel network was synthesized by a gelator of Zn(II)-Porphyrin-based **1 **(Figure 23) [213,214], stabilization of the gel network was achieved by the addition of fullerene.

**Figure 24.**
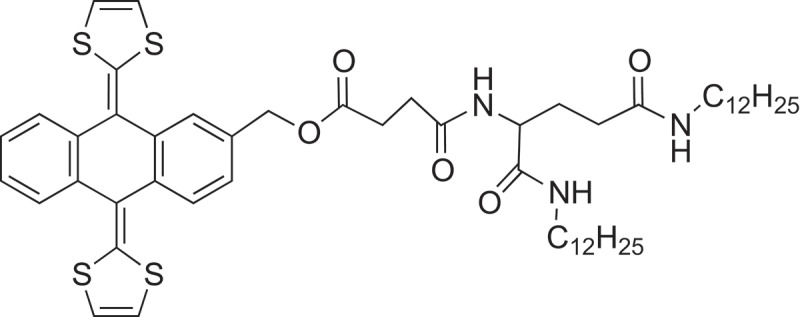
Gelator molecule 2.

Fullerene reinforces the gel matrix by forming a complex in host−guest fashion. It was reported that reinforcing the gel with fullerene remarkably enhanced both the thermal properties of the gel and its stability. Fullerene was also added to a gelator made by the addition of ex-tetrathiafulvalene-type to the lipid derivative of L-glutamide- base, **2** (Figure 24).

Improvement in the gelation capability of 2 was observed in polar organic solvents upon the addition of fullerene [215] This enhancement in gelation ability is due to the π−π stacking forces between the fullerene unit and the ex-tetrathiafulvalene unit as well as solvophobic interaction between the polar solvent and the gel; this overall result in, remarkable enhancement in the melting temperature of the gel.

## 5. Graphite

### 5.1. Background

**Figure 25.**
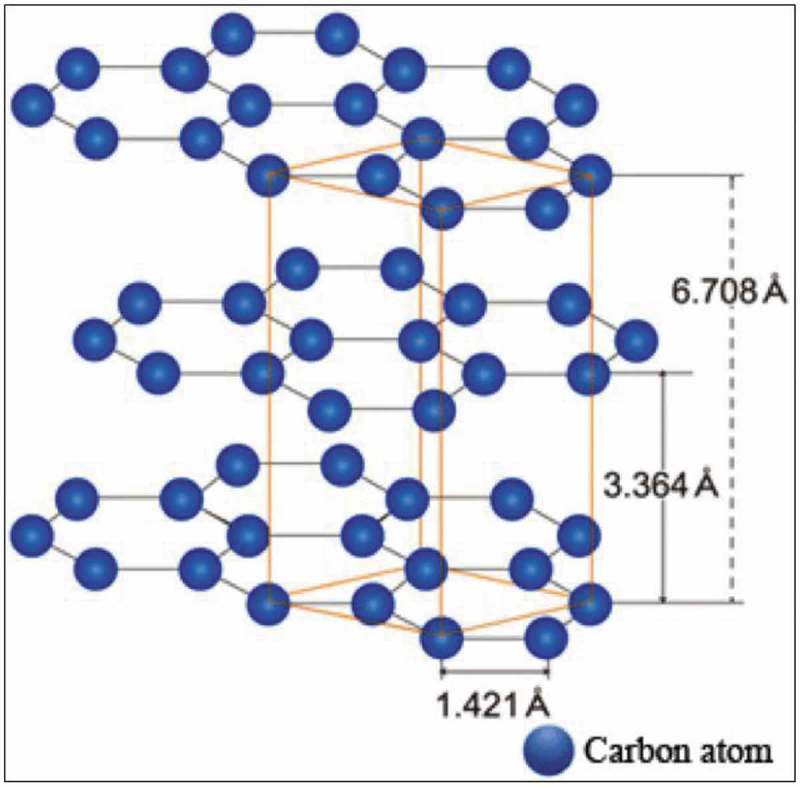
Structure of graphite.

Graphite is the most solid form of carbon beneath widespread situations (greater strong than diamond). Its structure is layered and planar, each layer being arranged in a hexagonal lattice Figure 25.

**Figure 26.**
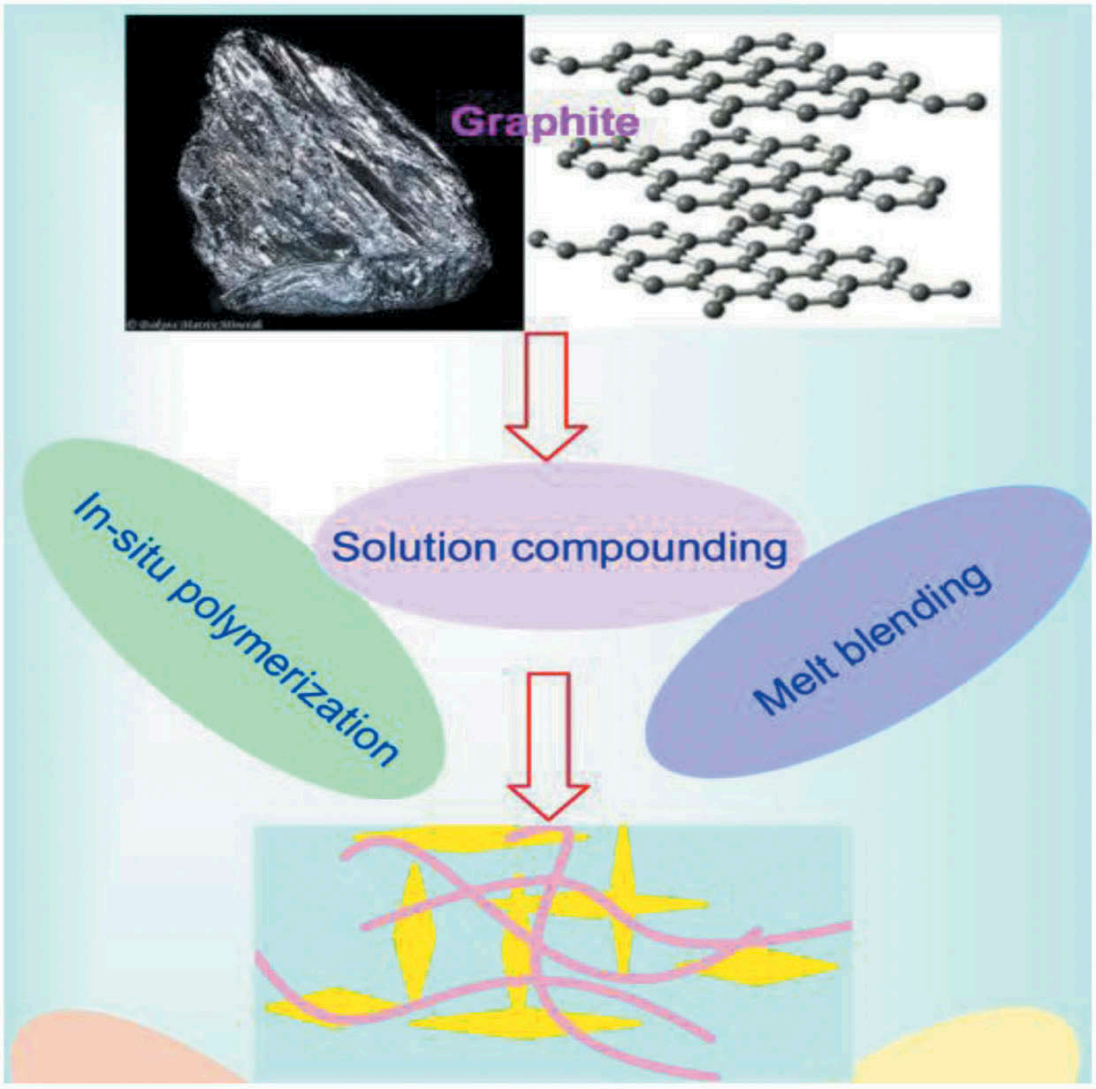
Polymer/graphite material.

In this assessment, we have reviewed the morphology fabrication strategies and different vital residences of graphite reinforced composites. The polymer/graphite nanocomposites had been fabricated by using solution blending, soften blending and *in situ* polymerization and diverse different methodologies. Figure 26.

### 5.2. Polymer nanocomposites based on graphite

**Figure 27.**
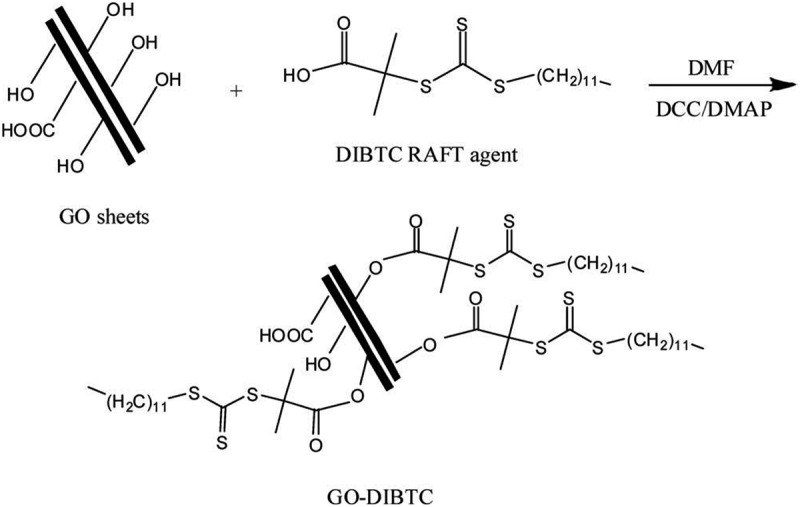
The overall synthesis route for the preparation of RAFT immobilized GO nanosheets (DMF: N, N-dimethylformamide, DCC: 1,3-dicyclohexyl carbodiimide, DMAP: 4-dimethylaminopyridine).

In latest years, diverse research have focused on the synthesis of polymer/graphite nanocomposites using chemically changed GO. In 2011, Hussein M. Etmimi et al. [216] suggested at the synthesis of polystyrene (ps)/graphite nanocomposites in miniemulsion polymerization. Graphite oxide (GO) become prepared and immobilized with dodecyl isobutyric acid trithiocarbonate (DIBTC) reversible addition-fragmentation chain switch (RAFT) agent. The hydroxyl groups of the cross have been attached to the DIBTC RAFT agent via an esterification process. The consequently modified cross turned into used for the preparation of polystyrene (playstation)/graphite nanocomposites in miniemulsion polymerization. The RAFT grafted GO (GO-DIBTC) at various loadings turned into dispersed in styrene monomer, and the resultant mixtures were sonicated inside the presence of a surfactant (sodium dodecylbenzene sulfonate) and a hydrophobe (hexadecane) to form miniemulsions. The strong miniemulsions therefore received have been polymerized the use of azobisisobutyronitrile as the initiator to yield encapsulated PS-GO nanocomposites as illustrated in Figure 27.

A similar procedure turned into used for the synthesis of the ps general by using miniemulsion polymerization. The oil section, including St, AIBN, DIBTC, and HD was combined with an aqueous solution of SDBS for 30 min. The mixture turned into then sonicated under the identical conditions for 15 min to achieve the miniemulsion. The polymerization was begun at 75°C and achieved for 10 h under a nitrogen atmosphere. Formulations used for the polymerization of PS-GO nanocomposites and the PS standard are tabulated in Table 4.

**Table 4.** Miniemulsion formulations used for the polymerization of PS-GO nanocomposites, and the PS standard.

Nanocomposite	DIBTC (g)	GO-DIBTC (g)	St (g)	AIBN (g)	SDBS/10 g of DDI Water (g)	HD (g)	DDI Water (g)
PS-Standard	0.0085	-	3.05	0.0052	0.060	0.15	50.18
PS-GO-1	-	0.030	3.01	0.0083	0.060	0.10	51.13
PS-GO-2	-	0.060	3.02	0.0080	0.060	0.10	50.17
PS-GO-3	-	0.091	3.06	0.0082	0.061	0.11	50.82
PS-GO-4	-	0.112	3.01	0.0080	0.062	0.12	50.30
PS-GO-5	-	0.137	3.08	0.0084	0.060	0.12	5042
PS-GO-6	-	0.170	3.06	0.0080	0.060	0.11	50.13
PS-GO-7	-	0.204	3.01	0.0081	0.062	0.14	50.23

The molar mass and PDI of ps inside the nanocomposites decreased markedly because the RAFT-functionalized GO concentration increased, as predicted for RAFT-mediated polymerization. TEM observations confirmed that the PS-GO nanocomposites had exfoliated morphology, even at notably high graphite loadings. TGA effects indicated that all PS-GO nanocomposites had better thermal stabilities than the neat PS. but, it was discovered that the thermal stability of the PS-GO nanocomposites is not a function of graphite attention (i.e., GO-DIBTC). A growth in modified GO content did now not have any impact on the thermal stability of the received nanocomposites. This was attributed to the effect of RAFT-grafted GO at the molar masses of the PS, which reduced considerably as the amount of GO-DIBTC inside the nanocomposites increased. moreover, the mechanical properties (i.e., storage and loss modulus) of the nanocomposites advanced appreciably as the amount of changed GO accelerated, as measured by way of DMA. The storage and loss modulus of the nanocomposites had been higher than those of the standard ps while the GO loadings reached 3% and 6%, respectively. However, because the modified GO content material increased inside the sample, a shift of the tan d peaks to decrease temperatures (i.e., lower g values) changed into record. This turned into attributed to the change in molar masses of the PS chains in the nanocomposites.

**Figure 28.**
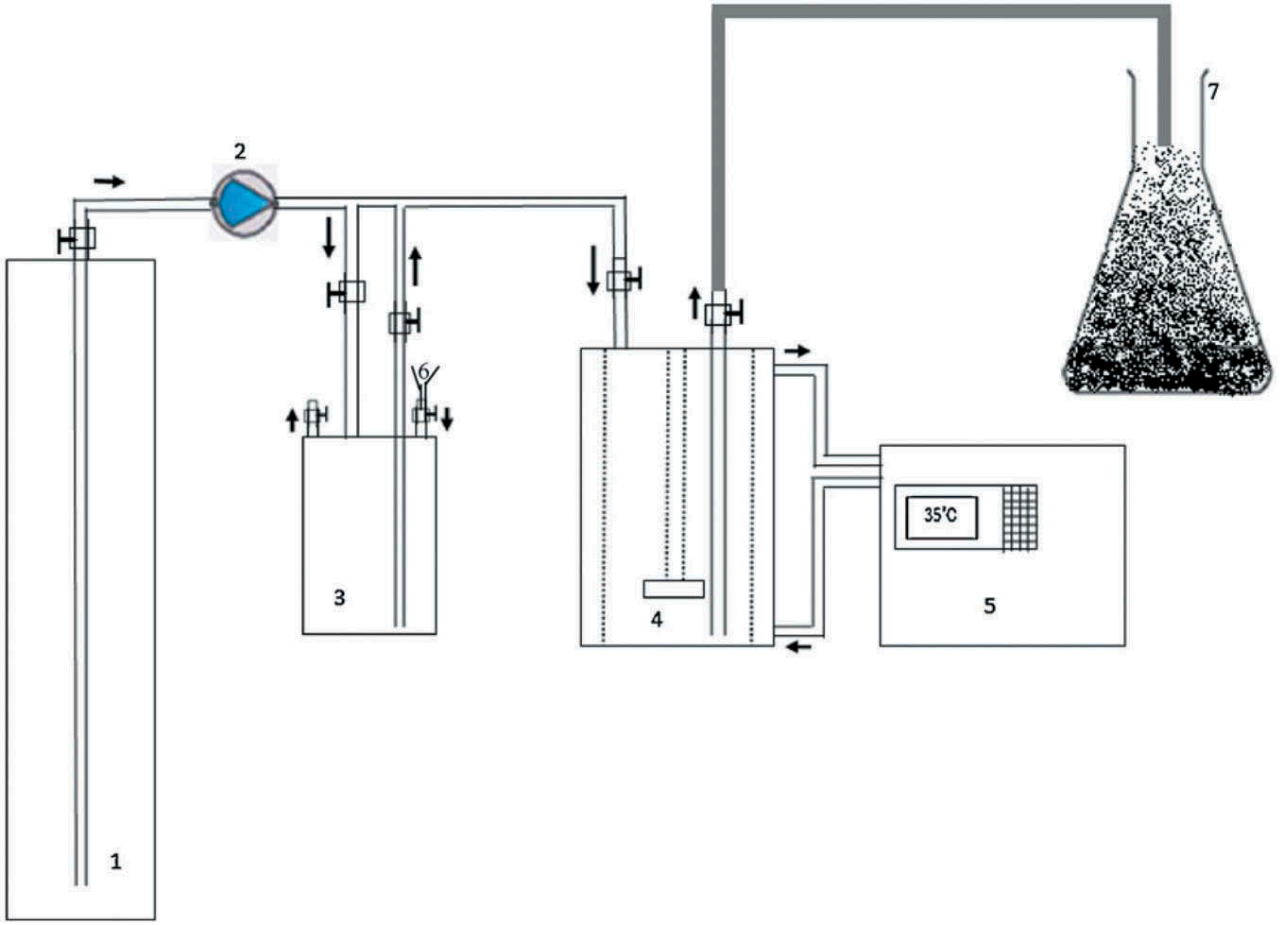
Schematic of the experimental setup, (1) CO2 cylinder, (2) high-pressure pump, (3) APS solution reservoir, (4) high-pressure autoclave, (5) water batch, (6) APS injection hopper, and (7) product accumulator.

Akbarinezhad and Sabourib [217] synthesized exfoliated PANI carbon nanocomposites with high electrical conduction via in situ polymerization in ScCO_2_ setting. Within the opening move, the ScCO_2_ medium was wont to expand carbon. Afterward, in place polymerization of aminoalkane monomers was performed in ScCO_2_/water interface to arrange exfoliated polyaniline carbon nanocomposites with dramatically higher electrical conduction than pristine polyaniline. Figure 28 shows the schematic experimental setup. For comparison, the higher than method was perennial in region conditions to arrange PGNs. The resulted nanocomposites were precipitated by adding the reaction mixture into 500 ml of HCl solution (0.2 M), followed by filtering and laundry mistreatment 50 ml methyl alcohol to get rid of unreacted monomers, oxidizer and byproducts. Finally, the powdery substance was repeatedly washed by HCl (0.2 M) solution and dried at 40 °C in vacuum for 24 h.

**Figure 29.**
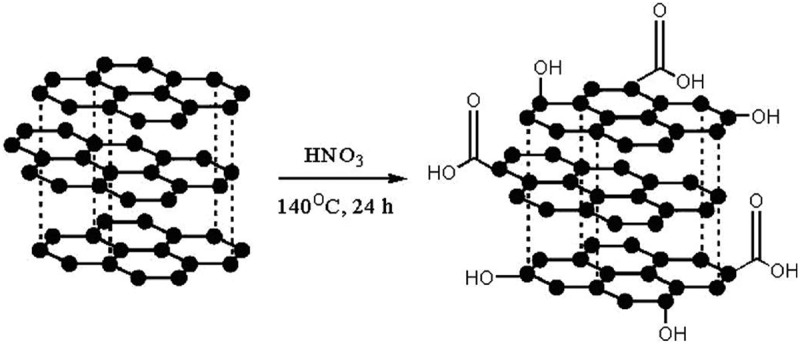
Schematic illustration of graphite structure modification.

The electrical conduction studies showed that the role of semiconductive expanded graphite in different weight percentages within the polymer matrix shows the improvement of the conduction of the conducting polyaniline. So, it is used ScCO_2_ as an alternate environmental friendly medium to prepare nanocomposites with superior properties. Sarat K. swain and Gyanaranjan Prusty [218] prepare polyacrylonitrile/graphite (PAN/EG) gas-barrier, ﬁre-retardant nanocomposites by employing an inexpensive green-emulsiﬁer-free emulsion technique. oxygen porosity decreases well with increased EG loading, that probably makes these materials suitable for packaging applications. Moreover, the results show that a nonconductive polymer becomes semiconducting with the addition of a tiny low amount of EG. PAN/EG nanocomposites are often used as transistors in nanoelectronic devices and packaging materials. Furthermore, Wanjun Liu [219] fabricate Exfoliate graphite nanoplatelets (xGnP) fortified polyamide 6 nanocomposites with extruder and injection decompose and characterized for their mechanical properties, morphology, and electrical resistance. The influence of melt compounding process on the physical properties of a polyamide 6 (PA6) nanocomposite strengthened with exfoliated graphite nanoplatelet (xGnP) has been studied. It was found that counter rotation (CNR) twins crew processed xGnP/PA6 nanocomposite had similar mechanical properties with co-rotation (CoR) twin screw processed or with CoR conducted with a screw style modified for nanoparticles (MCoR). Microscopic features showed that the CNR processed nanocomposite had higher xGnP dispersion than the (CoR) twin screw processed and changed screw (MCoR) processed ones. It had been additionally found that the CNR processed nanocomposite at a given xGnP content showed the lowest graphite X-ray diffraction peak at 26.5 indicating a higher xGnP dispersion within the nanocomposite. Additionally, it absolutely was additionally found that the electrical conductivity of the CNR processed 12 wt.% xGnP-PA6 nanocomposite is more than 10 times higher than the CoR and MCoR processed ones. These results indicate that higher dispersion of an xGnP-PA6 nanocomposite is possible in CNR twins crew process than standard CoR process. Moreover, Asima Naz1 et al. [220] fabrication of three material nanocomposites derived from non-functionalized and functionalized graphite, polyaniline (PANi), poly(styrene co-maleic anhydride) cumene terminated (PSMA) and 4,40-oxydianiline (ODA) via layer by layer *in situ* oxidative polymerization. A series of NF-G/PANi/PSMA/ODA and F -G/PANi/PSMA/ODA nanocomposites haven been a fabrication. First, the surface modification of graphite was dispensed by the treatment of puriﬁed graphite with HNO_3_ (see Figure 29).

**Figure 30.**
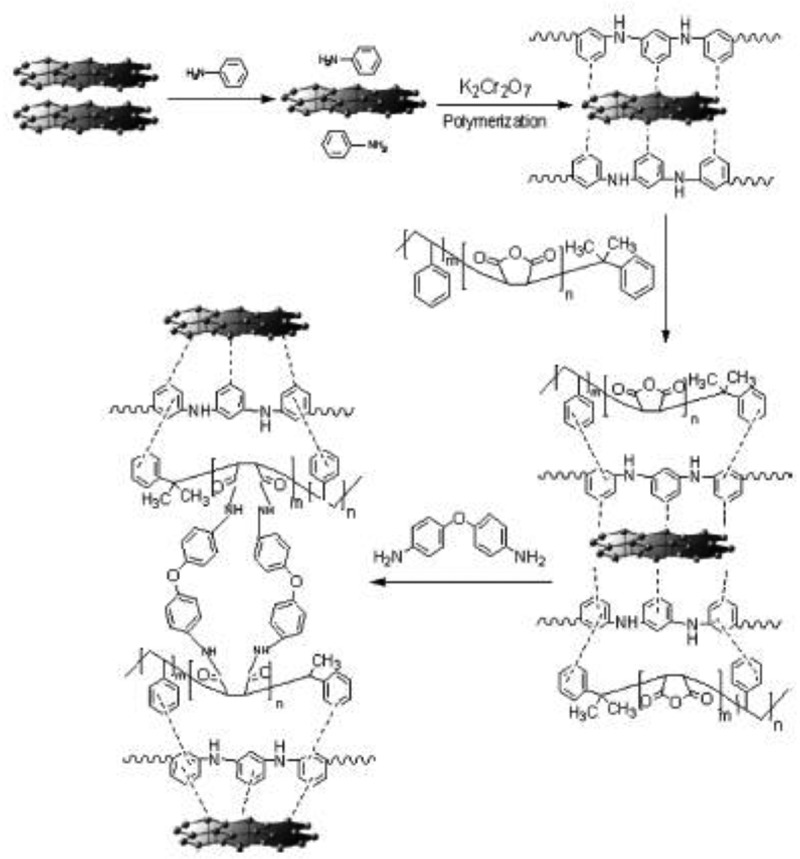
Schematic synthesis of G = PANi = PSMA = ODA.

After that bilayered nanocomposite, NF-G/PANi/PSMA and F-G/PANi/ PSMA were prepared. Then, the bi-layered nanocomposite was cross-linked mistreatment ODA through ring-opening polymerization of PSMA (Figure 30). To the most effective of our data, the core-shell NF-G and F-G/PANi/PSMA/ODA nanocomposites are with synthesized first time through this rout. It had been determined that surface medication of graphite has proficiently initiated the polymerization and time interval of the chemical compound chains within the graphite. The physical characteristics of resultant nanocomposites were explored mistreatment varied techniques. A colossal kind of potential uses for graphite nanocomposites developed from diamine cross-linked graphite could also be explored in the future.

In addition, LANG Jaroslav et al [221]. are targeted on study polypropylene (PP)/graphite composites wherever graphite served as filler for the PP matrix either in a pure state or functionalized by TiO_2_ and ZnO nanoparticles. The graphite and functionalized graphite content within the PP composites was 75 wt.%. The prepared samples were characterized with x-ray diffraction and Raman spectroscopy ways and also the Martens hardness of the material decided. The very best HM value was exhibited by a composite containing large grain graphite (denoted as PP/GRA1). The HM was littered with thermal treatment (170°C for 1 h). The HM of the composites containing graphite fillers raised, whereas just in case of the composites containing functionalized graphite fillers the decrease in HM was determined. Results summarized in Table 5 unconcealed that pure PP and composites containing GRA1 and GRA2 fillers showed an increase in Martens hardness (HM) once thermal treatment whereas just in case of composites containing photoactive fillers the HM values reduced.

**Table 5.** Comparison of Martens hardness (HM) values for samples before and after thermal treatment at 170°C for 1 h.

Sample	HM	
	25°C	170°C (1h)
PP	31	490
PP/GRA1	548	653
PP/GRA2	378	405
PP/GRA1Ti	191	146
PP/GRA2Zn	269	224

Recently set of novel nanocomposites supported graphite nanoplatelets (GnP) and poly(ethylene terephthalate) (PET) was synthesized by Qiushu Xu, et al. [222] via in situ polymerization by soften spinning technique. The homogenous PET matrix nanocomposites containing the content of value below 2 were generated because of the application of preliminary PVP-K30 treatment to GnP/EG suspensions and soften compounding throughout the *in situ* polymerization. As featured with smart heat conductivity and thermal diffusivity.

## 6. Biopolymer-based CNT and/or graphene nanocomposites

### 6.1. Background

**Figure 31.**
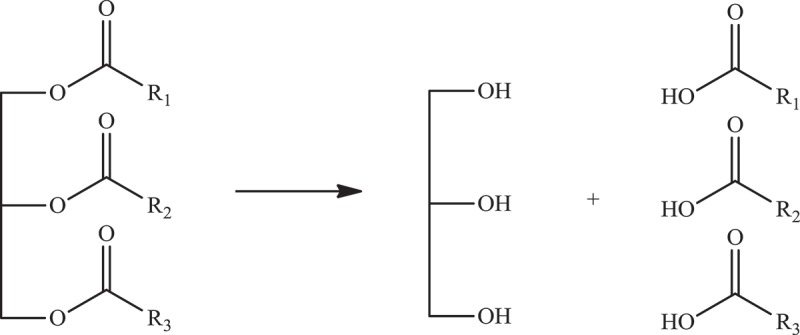
Structure of triglycerides.

A sustainable and more environmental-friendly alternative to synthetic polymers are vegetable oils. Vegetable oils are an excellent source of starting materials for building new polymers as they are biologically friendly, relatively low cost, and commercially available. They are also labile as they include multifunctional groups, such as epoxy, carbon-carbon double bonds and hydroxyl groups, which can be further modified to obtain different functional groups. There are many plants sources to extract vegetable oils from such as soybean, palm tree, and castor. Figure 31 shows the chemical structure and formula of some common vegetable oils. In general, vegetable oils are triglycerides, which are essentially three chains of fatty acid that is attached to the glycerol by an ester linkage. Table 6 illustrates a variety of common fatty acids in vegetable oils.

**Table 6.** Variety of common fatty acids in vegetable oils.

Fatty Acid	Formula	Structure
Caprylic	C_8_H_16_O_2_	
Capric	C_10_H_20_O_2_	
Lauric	C_12_H_24_O_2_	
Myristic	C_14_H_28_O_2_	
Palmitic	C_16_H_32_O_2_	
Palmitoleic	C_16_H_30_O_2_	
Searic	C_18_H_36_O_2_	
Oleic	C_18_H_34_O_2_	
Liuoleic	C_18_H_32_O_2_	
Linolenic	C_18_H_30_O_2_	
α-Elcoslearic	C_18_H_30_O_2_	
Ricinoleic	C_18_H_34_O_3_	
Vernolic	C_18_H_32_O_3_

### 6.2. Biopolymer-based nanocomposites

**Figure 32.**
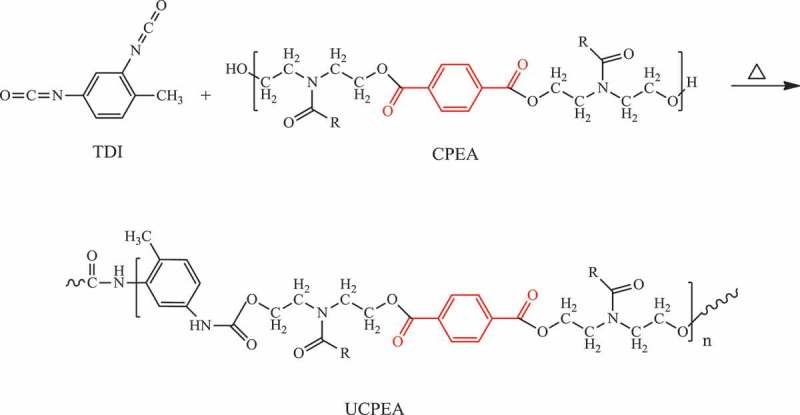
Synthesis of UCPEA.

Castor oil is a cheap renewable resource for the manufacture of environmental friendly resins [223]. Polymers synthesized from castor oil are recyclable, low cost and easy to alter [224]. Castor oil is used for the synthesis of many polymers such as polyesteramide, polyurethane, and mixed with commercial polymethylmethacrylate, polystyrene, polyvinyl alcohol [225]. Moreover, the presence of 12-hydroxy-9-cis-octadecenoic acid (ricinoleic acid) and a hydroxyl group in castor oil facilitate fabrication of different polyesters and polyester-anhydrides. The dangling chains in CO provide hydrophobicity influencing the physical and mechanical properties of polyurethane (PU). Ali at el. reported the synthesis of new COPUs-MWCNTs polymer nanocomposite based on green and renewable castor oil as a polyol source and further reinforced with purified MWCNTs [226]. Recently, a solvent-free method for the preparation of polyol from castor oil based fatty acid (COFA) and epoxidized soybean oil was reported [227]. Condensation polymerization method for the preparation of castor oil based polyesteramide (CPEA) resin was reported using of N-N-bis (2-hydroxyethyl) castor oil fatty amide (HECA) and terephthalic acid and was further modified with different percentages of toluene-2,4-diisocyanate (TDI) (7, 9, 11, and 13) wt.% to obtain poly(urethane-esteramide) (UCPEA), through addition polymerization. The UCPEA polymers were reinforced by TiO_2_ nanoparticles (0.1, 0.2, 0.3, 0.4, and 0.5 wt%) (see Figure 32).

The new materials after the addition of the fillers revealed promising antimicrobial properties [228]. A very promising potential biopolymer source to fossil fuels is soy protein isolate (SPI). Recently, Jin et al. reported a new synergistic reinforcing motif that might open a new pathway for constructing high-performance nanocomposites [229]. The route to assemble the high-performance SPI nanocomposite films reinforced by CNT and graphene nanosheets and nanofibrillated cellulose (NFC) by thecasting method. The use of web-like nanofibrillated cellulose helps uniform dispersion of graphene/CNTs in the biopolymer matrix. Moreover, the NFC allows the high extent of cross-linkage combination between the fillers and SPI matrix. As result of introducing nanocomposites the tensile strength of SPI/G/CNT/NFC film considerably increased from 31.76% in neat SPI film to 78.9% also, the water vapor permeability decreased after introduction of G/CNT/NFC to SPI [229].

## 6. Conclusions

The interest in polymer nanocomposites based on carbon fillers will continue for the formation of advanced materials. The fascinating features of carbon-based polymer nanocomposites will aspire chemists in tailoring novel polymers with predesigned chemical structures to ease their fabrication and solubility in organic solvents as well as to be suitable for compounding with specific carbon-based nanomaterials. The review emphasized mainly CNT preparation and composite formation with different polymers in addition to other carbon-based materials such as graphene, fullerene, and graphite. It is believed that this review may inspire the scientific community in exploiting the synergism of nano-fillers in polymer composites aiming at discovering new materials.
